# Circulating microRNA signatures of cachexia and cancer in *Canis familiaris* as a comparative oncology model for human disease

**DOI:** 10.1002/1878-0261.70293

**Published:** 2026-07-22

**Authors:** Soon‐Seok Park, Kyongman An, Gyeonghwa Kim, Soo‐Nyun Choi, Hong‐Ki Lee, Keun Hur, Kyu‐Shik Jeong

**Affiliations:** ^1^ College of Veterinary Medicine Kyungpook National University Daegu City Republic of Korea; ^2^ AI‐Bio Convergence Research Institute, Department of Industrial AI Engineering, Graduate School of Management of Technology Hoseo University Asan City Republic of Korea; ^3^ Department of Medical Science, College of Medicine CHA University Seongnam City Republic of Korea; ^4^ Department of Biochemistry and Cell Biology, School of Medicine Kyungpook National University Daegu City Republic of Korea; ^5^ EHL Bio Co., LTD Uiwang City Republic of Korea; ^6^ Department of Pet Industry, Adventure College Daegu Haany University Gyeongsan City Republic of Korea; ^7^ Stellamed Co., LTD Daegu City Republic of Korea

**Keywords:** biomarker, cachexia, canine, comparative oncology, microRNA, sarcopenia

## Abstract

Cachexia, a severe muscle‐wasting syndrome, lacks objective biomarkers for early detection. This study aimed to identify circulating microRNA signatures for cachexia and cancer in senior dogs, a comparative oncology model for human disease. Serum microRNA expression was quantified in a cohort of 25 dogs, clinically classified by cachexia and cancer status. We identified a distinct signature for cachexia, with significant downregulation of miR‐15a, miR‐15b, miR‐16, and miR‐140. Circulating miR‐16 emerged as the most robust individual biomarker for cachexia (AUC = 0.899). Furthermore, a sex‐specific analysis revealed that miR‐140 was significantly lower in female dogs with cancer compared to noncancer controls (*P* = 0.045), consistent with a sex‐ and disease‐specific dual suppression mechanism involving estrogen receptor signaling and cachexia‐driven systemic inflammation (corrected AUC = 0.667). These findings suggest circulating microRNAs as promising, noninvasive biomarkers for canine cachexia and highlight miR‐140 as a candidate sex‐specific biomarker for female cancers, warranting validation in larger cohorts. This work reinforces the value of the domestic dog as a spontaneous model for translational biomarker discovery.

## Graphical abstract

Circulating microRNAs as biomarkers of cachexia and sex‐specific cancer in senior dogs. In 25 client‐owned dogs, four circulating miRNAs (miR‐15a, miR‐15b, miR‐16, miR‐140) were downregulated in cachexia, with miR‐16 the strongest individual biomarker (AUC = 0.899). Among female dogs, miR‐140 was further suppressed in cancer subjects (*P* = 0.045), consistent with a dual‐hit mechanism combining cachexia‐driven inflammation and estrogen receptor signaling.

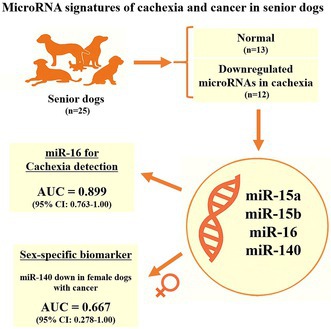

**Abbreviations**
AUC: area under the curve
BCS: body condition score
BW: body weight
CI: confidence interval
EPV: events‐per‐variable
ER*α*
: estrogen receptor alpha
IACUC: Institutional Animal Care and Use Committee
IGF‐1: insulin‐like growth factor 1
IL‐1*β*
: interleukin‐1 beta
IL‐6: interleukin‐6
IRS1: insulin receptor substrate 1
MCS: muscle condition score
miRNA: microRNA
ND: not detected
qRT‐PCR: quantitative reverse transcription polymerase chain reaction
ROC: receiver operating characteristic
RTN4: reticulon 4 (gene encoding Nogo‐A)
SEM: standard error of the mean
TNF‐*α*
: tumor necrosis factor‐alpha
UTR: untranslated region

## 1 Introduction

Cachexia is a complex and multifactorial metabolic syndrome defined by a severe and progressive loss of skeletal muscle mass, with or without a concurrent loss of fat mass, that cannot be fully reversed by conventional nutritional support [1, 2]. According to the international consensus definition, cancer cachexia is driven by a combination of reduced food intake and abnormal metabolism, resulting in a negative protein and energy balance [1, 2, 3]. This profound muscle wasting is distinct from simple starvation or age‐related muscle loss (sarcopenia) and is propelled by a systemic inflammatory response and complex metabolic derangements that shift the body into a hyper‐catabolic state [1, 2, 4]. It is a devastating comorbidity of many chronic diseases, most notably cancer, where it is estimated that over 50% of human patients experience unintentional weight loss [1]. The clinical consequences are severe, leading to reduced quality of life, diminished immune function, impaired response to therapies, and increased mortality; indeed, cachexia is directly responsible for over 20% of cancer‐related deaths in humans [1, 2, 4].

The diagnosis of cachexia, particularly in its early stages, remains a significant clinical challenge in both human and veterinary medicine. Current methods often rely on tracking total body weight loss and subjective assessments like the body condition score (BCS) or muscle condition score (MCS) [1, 3]. These methods are often insensitive to the early loss of lean body mass, which can be masked by concurrent fat gain or fluid retention, a condition known as sarcopenic obesity [5]. This diagnostic delay prevents timely intervention until the syndrome is advanced and often irreversible [1, 3, 5]. This highlights a critical unmet need for objective, sensitive, and noninvasive biomarkers for the early detection of muscle wasting.

Circulating microRNAs (miRNAs) have emerged as a promising class of such biomarkers [6]. MiRNAs are a class of small, noncoding RNA molecules, typically 19–24 nucleotides in length, that function as critical post‐transcriptional regulators of gene expression [7]. By binding to the 3′ untranslated regions (UTRs) of target messenger RNAs (mRNAs), they primarily induce mRNA degradation or translational repression, thereby fine‐tuning vast gene networks [7]. A single miRNA can regulate hundreds of target genes, giving them profound biological impact on processes such as proliferation, apoptosis, differentiation, and inflammation [7]. Critically for biomarker development, miRNAs are remarkably stable in various body fluids, including serum and plasma, where they are protected from degradation by being packaged within extracellular vesicles or bound to proteins [6, 8]. This stability, combined with their often tissue‐ and disease‐specific expression patterns, makes them highly promising candidates for minimally invasive biomarkers for a spectrum of complex diseases, including cancer and its systemic complications in both humans and dogs [9, 10, 11].

The domestic dog (*Canis familiaris*) serves as an invaluable spontaneous model for studying human muscle‐wasting syndromes. The field of comparative oncology leverages the similarities between naturally occurring cancers in pets and humans to accelerate therapeutic development [12, 13, 14]. Companion dogs share the human environment, have a significantly longer lifespan than laboratory rodents, and develop cancers that closely parallel their human counterparts in genetics, biology, and clinical progression [12, 13, 14]. Cancer cachexia is a frequent and clinically significant complication in veterinary oncology, contributing to poor outcomes similar to those seen in human patients [15]. Therefore, identifying biomarkers in this population can provide powerful translational insights.

The selection of candidate miRNAs for this study was guided by a two‐tiered, hypothesis‐driven approach. As the first tier, a bioinformatic miRNA target prediction analysis was performed against the regulatory network of Nogo‐A (RTN4), a transmembrane endoplasmic reticulum‐resident protein markedly upregulated in skeletal muscle under conditions of severe wasting, including amyotrophic lateral sclerosis [16] and DMD‐associated myopathies [17]. Because Nogo‐A itself is not detectable in serum, this screen was not designed to measure Nogo‐A directly; rather, it identified miRNAs that regulate RTN4 expression in muscle tissue and are released systemically from remodeling muscle into circulation via extracellular vesicles [6, 18, 19]. The top‐ranked predicted regulators of the human RTN4 gene, hsa‐miR‐16‐5p, hsa‐miR‐15b‐5p, hsa‐miR‐152‐3p, hsa‐miR‐195‐5p, and hsa‐miR‐497‐3p, are tabulated in Table S1. As the second tier, miR‐15a and miR‐140 were added to the panel based on their independently established roles in cancer biology and muscle physiology. The miR‐15/16 family are canonical tumor suppressors targeting BCL2 [20, 21], regulators of systemic immune homeostasis [22], and direct modulators of the IGF‐1/PI3K/AKT anabolic axis via the LncIRS1/IRS1 pathway [23], mechanisms with direct relevance to the pathophysiology of cancer cachexia. miR‐140 is a transcriptional target of estrogen receptor alpha (ER*α*) signaling [24] and a regulator of muscle satellite cell proliferation [25], positioning it as a biologically plausible biomarker for both hormone‐sensitive cancers and muscle wasting. The Nogo‐A network screen thus served as the initial muscle‐pathology‐anchored selection rationale; the independent biology of each miRNA constitutes the primary scientific justification for the panel.

Therefore, the primary aim of this study was reframed to investigate this biologically potent miRNA signature in a cohort of companion dogs with naturally occurring cachexia and cancer. We sought to: (1) evaluate the diagnostic performance of circulating miR‐15b, miR‐16, and miR‐140‐5p for cachexia; (2) assess their association with cancer status; and (3) interpret their dysregulation within the robust biological context of their established roles as key regulators of inflammation, tumor suppression, and myogenesis, leveraging the unique translational power of the canine comparative oncology model.

## 2 Materials and methods

### 2.1 Ethical statement for animal studies

This study was conducted using serum from residual blood samples collected from client‐owned dogs presented to the Department of Veterinary Pathology, College of Veterinary Medicine at Kyungpook National University for diagnostic or therapeutic purposes. No new animal interventions were performed specifically for this study. The Institutional Animal Care and Use Committee (IACUC) of Kyungpook National University was consulted regarding the ethical oversight requirements for this study. All animal procedures were performed in accordance with the guidelines of the Institutional Animal Care and Use Committee (IACUC) and the IACUC determined that formal IACUC approval was not required, as the study involved only retrospective analysis of residual clinical specimens with no experimental procedures performed on animals. Written informed consent for the research use of residual samples and associated medical records was obtained from all dog owners prior to sample collection and analysis. All procedures involving animals were conducted in accordance with applicable animal welfare guidelines and institutional policies of Kyungpook National University.

### 2.2 Patient cohort and clinical classification

A total of 25 client‐owned domestic dogs (*Canis familiaris*), ranging in age from 7 to 17 years, were enrolled in this study. Upon presentation, each dog underwent a thorough physical examination, and a detailed clinical history was obtained from the owner. The dogs were classified into two primary groups based on vitality, lean body mass, BCS, and severity of the disease (Table S2). The Cachexia group (*n* = 12) was defined by clinical evidence of muscle wasting, assessed by palpation of muscle mass over the temporal bones, scapulae, and lumbar vertebrae, accompanied by signs of weakness or infirmity in the context of an underlying chronic disease. The normal group (*n* = 13) consisted of dogs that did not exhibit these signs of muscle loss or weakness. The cohort was further categorized based on a definitive diagnosis of cancer, established through diagnostic imaging (radiography, ultrasonography, CT, or MRI) and/or histopathology. This resulted in a cancer group (*n* = 14) and a noncancer group (*n* = 11). The noncancer group included dogs with various conditions such as orthopedic or neurological diseases. Detailed clinicopathological data for each patient are provided in Table S3.

The clinical classification of cachexia in this study was based on three concordant lines of evidence: (1) a detailed clinical history documenting involuntary body weight loss and reduced food intake as reported by the owner; (2) physical examination by a board‐certified veterinarian, including palpation of muscle mass over the temporal bones, scapulae, and lumbar vertebrae, assessed using a MCS framework; and (3) supportive radiographic evidence of epaxial muscle atrophy on lateral thoracic radiographs (see Section 3.3). This multimodal approach is consistent with veterinary cachexia consensus criteria [15]. Importantly, cachexia was distinguished from simple aging‐related sarcopenia by the requirement for an underlying chronic disease driving the catabolic state, consistent with the Fearon et al. international consensus definition [1].

Where available, serum clinical laboratory values (albumin, hemoglobin, total protein) were extracted from the dogs' medical records. The availability of these values was variable across the retrospective cohort; complete laboratory panels were available for 18/25 dogs. The overall pattern of clinical laboratory findings, summarized in Table S2, was consistent with the inflammatory‐catabolic phenotype of cancer‐associated cachexia: the cachexia group showed a trend toward lower albumin (mean 2.8 ± 0.4 g/dL) compared to the normal group (mean 3.1 ± 0.5 g/dL), and lower hemoglobin (mean 11.2 ± 2.1 g/dL vs. 14.8 ± 2.8 g/dL), consistent with hypoalbuminemia and anemia of chronic disease observed in human cachexia [1, 26].

Tumor types for all cancer‐positive dogs were confirmed by histopathology and/or advanced diagnostic imaging. Among the 14 cancer dogs, 9 presented with tumors of the mammary gland, reproductive tract, or perianal region (predominantly hormone‐sensitive tumor types), 3 had splenic or soft tissue sarcomas (including one hemangiosarcoma [C31] that received adjuvant chemotherapy), and 2 had other tumor types (prostate tumor [C8]; oral melanoma [C23]). Only one dog (C31) received chemotherapy during or prior to the study period.

Two dogs in the cachexia group warrant explicit mention regarding pathophysiological heterogeneity. C33 (12‐year‐old castrated male Maltese) was classified as cachexia based on documented muscle wasting, progressive anorexia, and weight loss; however, the underlying diagnosis was hypoadrenalism (Addison's disease), a condition in which muscle loss and weakness arise from glucocorticoid/mineralocorticoid deficiency rather than the pro‐inflammatory‐catabolic cascade that defines cancer‐associated cachexia. C22 (16‐year‐old intact male Shih Tzu) presented with cerebral infarction, severe brain atrophy, and cognitive dysfunction; his muscle wasting reflects neurological disuse rather than systemic inflammatory catabolism. Both dogs met the clinical criteria applied for group assignment. Their inclusion represents a recognized limitation of retrospective cohort design; their distinct pathophysiological mechanisms may introduce heterogeneity into the cachexia group miRNA signature and are explicitly acknowledged.

### 2.3 Radiographic assessment of muscle mass

**Fig. 1:**
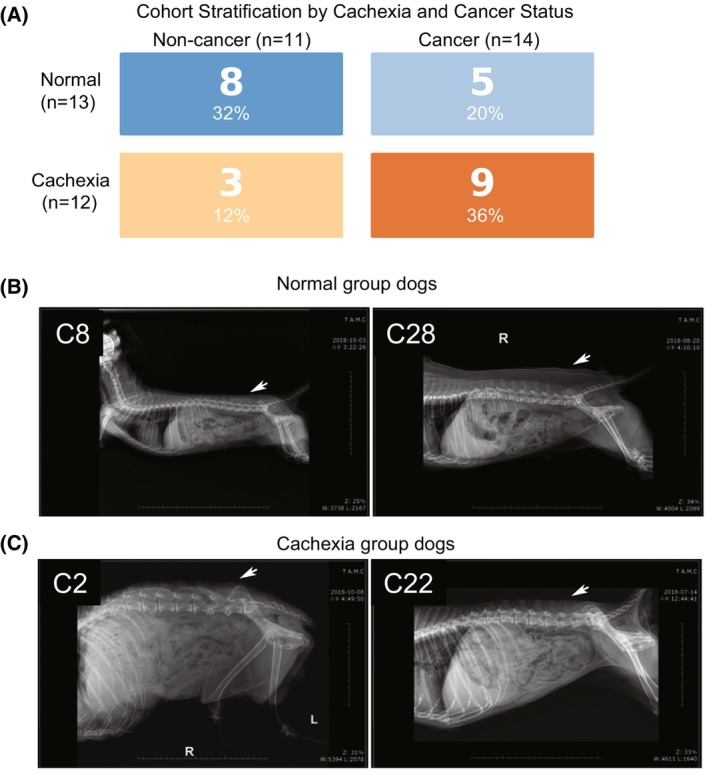
Characterization of the canine cohort and radiographic phenotype of cachexia. (A) 2 × 2 contingency table illustrating the stratification of the 25‐dog cohort by cachexia and cancer status, showing the overlap between clinical groups. The high prevalence of cancer in the cachexia group (9/12; 75%) compared with the normal group (5/13; 38%) is highlighted. (B, C) Representative lateral thoracic radiographs. (B) A dog from the normal group showing substantial epaxial muscle mass dorsal to the spine (arrow). (C) A dog from the cachexia group showing marked muscle atrophy, indicated by the reduced space between the dorsal spinous processes and the skin (arrow). These images are representative clinical radiographs intended for qualitative morphological assessment rather than absolute quantitative measurement.

Patient examinations were assessed via X‐ray (Ecoray Co., Ltd, USA), ultrasound (Shenzhen Mindray, Model DC‐8, USA), MRI (Toshiba, Excelart Vantage 1.5 T MRI), and CT (Toshiba, Aquilion Lightning, 16‐row helical CT). To complement the subjective muscle condition scoring with an objective measure, we examined lateral thoracic radiographs of the dogs for evidence of muscle loss. In each radiograph, the depth of the epaxial muscles (the longissimus dorsi and associated musculature) dorsal to the thoracic spine was assessed. In healthy, well‐muscled dogs, there is a substantial soft tissue thickness over the dorsal spinous processes, creating a clear separation between the bony spine and the skin line. In dogs with severe muscle atrophy, this soft tissue layer is markedly thinned. For this study, radiographs of the thorax (when available from diagnostic workups) were reviewed blindly by a veterinary radiologist. A representative radiograph from a normal dog (with robust epaxial musculature) and from a cachectic dog (with obvious epaxial muscle loss) is presented in Fig. 1. The radiographic findings were used qualitatively to support the clinical classification of cachexia: a narrowed dorsal muscle layer and protruding vertebral spinous processes on X‐ray corresponded to advanced muscle wasting.

### 2.4 Serum collection and processing

Venous blood (2–5 mL) was collected from each dog via jugular or cephalic venipuncture, using standard clot‐activator vacutainer tubes. Samples were allowed to clot at room temperature for ~30 min, then centrifuged at 2000 × g for 10 min at 4 °C. The resulting serum was carefully separated and aliquoted into RNase‐free tubes. All serum samples were immediately stored at −80 °C until analysis to preserve RNA integrity. Hemolyzed samples (which can release intracellular miRNAs) were avoided or excluded to prevent confounding elevations of miRNAs not truly circulating.

### 2.5 Circulating miRNA extraction and quantification

Total circulating miRNA was extracted from 200 μL of serum using the miRNeasy Serum/Plasma Kit (Qiagen, Hilden, Germany) according to the manufacturer's protocol. The concentration and purity of the extracted RNA were measured using a NanoDrop ND‐1000 spectrophotometer (Thermo Fisher Scientific, Waltham, MA, USA). For normalization of sample‐to‐sample variation in RNA extraction and subsequent amplification steps, a synthetic *Caenorhabditis elegans* miRNA not present in mammals, cel‐miR‐39, was spiked into each sample during the lysis step. For cDNA synthesis, reverse transcription was performed on 50 ng of total RNA using the TaqMan MicroRNA Reverse Transcription Kit (Applied Biosystems, Foster City, CA, USA). Quantitative real‐time PCR (qRT‐PCR) was performed using SYBR Green PCR Master Mix (Applied Biosystems) on a real‐time PCR system. Customized primers for nine candidate canine miRNAs (miR‐15a, miR‐15b, miR‐16, miR‐140, miR‐148a, miR‐148b, miR‐152, miR‐195, and miR‐497), selected via the two‐tiered bioinformatic and biology‐driven rationale described in the Introduction (see Table S1 for the full Nogo‐A/RTN4 target prediction ranking and *a priori* additions), were synthesized (Bionics, Seoul, Korea). All reactions were performed in triplicate. The relative expression of each miRNA was calculated using the 2^−ΔΔCt^ method, with normalization to the exogenous spike‐in control, cel‐miR‐39. In brief, ΔCt = Ct(miRNA)–Ct(cel‐miR‐39), and ΔΔCt for each target miRNA was obtained by subtracting the ΔCt of a calibrator sample (e.g., the average of healthy controls). Fold change in expression relative to calibrator = 2^−ΔΔCt^.

### 2.6 Serum cytokine and growth factor quantification

Serum concentrations of interleukin‐6 (IL‐6) and insulin‐like growth factor‐1 (IGF‐1) were measured in all 25 dogs using commercially available canine‐specific ELISA kits. For IL‐6, absorbance values (OD450–OD540 nm) were measured using a sandwich ELISA (standard curve range: 0–1500 U/mL). For IGF‐1, serum concentrations were quantified in ng/mL using a standard curve with the following parameters: standard range 0–1500 ng/mL, with a conversion factor of 22 for the dilution used. All assays were performed in duplicate; samples with > 15% coefficient of variation were repeated. One dog (C02, spayed female Maltese with active bilateral ovarian cysts at the time of blood collection) had an IGF‐1 value of 256.5 ng/mL, more than 15 standard deviations above the cohort mean. As this extreme elevation is attributable to ovarian cyst‐driven hormonal upregulation of IGF‐1 rather than a cachexia‐related process, C02's IGF‐1 value was excluded from group comparison analyses, while all other data for this dog were retained. ND (not detected) values for IGF‐1 were assigned where the assay returned values below the lower limit of detection. One dog (C28, 9‐year‐old castrated male Pomeranian with neurogenic lameness) exhibited an anomalously elevated serum miR‐195 value (normalized expression: 0.570), representing a z‐score of +4.2 standard deviations above the cohort mean (excluding C28: mean = 0.059). This extreme elevation, in the absence of any plausible biological cause, is consistent with a hemolysis artifact or technical variation during serum preparation. C28 was retained in all analyses to avoid post hoc sample exclusion bias.

### 2.7 Receiver operating characteristic (ROC) curve analysis

To evaluate the diagnostic performance of individual miRNAs and the multi‐miRNA panel, ROC curve analysis was conducted using graphpad Prism (Version 5.0, graphpad Software, San Diego, CA, USA). For a four‐miRNA cachexia panel, comprising miR‐15a, miR‐15b, miR‐16, and miR‐140, selected based on their significant differential expression established in Results Section 3.3, a composite biomarker score was first generated for each subject. This was achieved by performing a binary logistic regression with cachexia status as the dependent variable and the expression levels of miR‐15a, miR‐15b, miR‐16, and miR‐140 as covariates. The resulting predictive probabilities from this model were then used as the input variable for the ROC analysis. For the single‐miRNA analysis (miR‐140 in the female cancer cohort), the relative expression values (calculated as 2^−ΔCt^) served directly as the test variable.

The Area Under the Curve (AUC), along with its 95% confidence interval (CI), was calculated to quantify the overall ability of the biomarker(s) to discriminate between clinical groups (e.g., cachexia vs. normal; cancer vs. noncancer). The *P*‐value associated with the AUC was calculated to test the null hypothesis that the AUC was equal to 0.5 (i.e., the model has no discriminatory ability better than chance). The optimal diagnostic cutoff threshold on the ROC curve, which maximizes the trade‐off between sensitivity and specificity, was determined using the Youden's J index (J = sensitivity + specificity −1). The sensitivity and specificity at this optimal cutoff point were reported.

### 2.8 Statistical analysis

All statistical analyses were conducted using graphpad Prism software (Version 5.0, graphpad Software, San Diego, CA, USA). Data were assessed for normal distribution. Comparisons of miRNA expression levels between two independent groups (e.g., normal vs. cachexia; cancer vs. noncancer) were performed using an unpaired Student's *t*‐test. A *P*‐value of less than 0.05 was considered statistically significant. All data are expressed as mean ± standard error of the mean (SEM). To evaluate the diagnostic performance of the significantly altered miRNAs, ROC curve analysis was performed. The AUC, along with its 95% CI, was calculated to assess the ability of the miRNAs to discriminate between clinical groups. To formally evaluate whether age confounded the association between cachexia status and miR‐16 expression, a multiple linear regression model was constructed with miR‐16 expression as the dependent variable and both age (continuous, in years) and cachexia status (binary: 0 = normal, 1 = cachexia) as simultaneous independent variables (*n* = 25). Parameter estimates (*β*), standard errors, t‐statistics, and *P*‐values were calculated using ordinary least squares. Additionally, an independent‐samples *t*‐test (with Levene's test for equality of variances) was used to compare the mean age between the normal and cachexia groups. A Spearman rank‐order correlation was performed to assess the relationship between age and each of the nine miRNAs across the full cohort.

## 3 Results

### 3.1 Clinicopathological characteristics of the canine cohort

The study cohort consisted of 25 senior dogs of various breeds, with ages ranging from 7 to 17 years (Table S3). The cohort was stratified based on the presence of cachexia and cancer into a normal group (*n* = 13) and a cachexia group (*n* = 12), and concurrently into a noncancer group (*n* = 11) and a cancer group (*n* = 14) (Fig. 1A). A detailed statistical breakdown of the cohort was performed to compare the normal and cachexia groups across key demographic variables, including age, sex, and body weight, as summarized in Table 1. The groups appeared to be reasonably well‐matched with the mean age of the normal group was 11.8 ± 2.8 years, while the cachexia group was slightly older with a mean age of 13.3 ± 3.1 years. This difference is unlikely to be a major confounder, as both groups consist of geriatric patients. Similarly, the mean body weights were comparable (6.2 ± 2.4 kg for the normal group vs. 5.7 ± 2.3 kg for the cachexia group). This overlap in absolute body weight reinforces the concept that cachexia is defined by the process of involuntary lean mass loss, not simply by a low body weight, further highlighting the need for more sophisticated diagnostic markers. The sex distribution was also balanced between the groups. A key feature of the cohort was the unequal distribution of cancer between the primary clinical groups; 75% (9/12) of dogs in the cachexia group had a diagnosis of cancer, compared to only 38% (5/13) in the normal group. Further medical record analysis revealed clinical laboratory findings broadly consistent with the inflammatory‐catabolic phenotype of cancer cachexia. The cachexia group showed lower mean albumin (2.8 ± 0.4 g/dL vs. 3.1 ± 0.5 g/dL in the normal group) and hemoglobin (11.2 ± 2.1 vs. 14.8 ± 2.8 g/dL) values, consistent with hypoalbuminemia and anemia of chronic disease, hallmarks of the systemic inflammatory state described in the Fearon et al. consensus definition [1]. Serum IGF‐1 concentrations, measured by ELISA, were significantly elevated in the cachexia group (19.31 ± 4.39 ng/mL; *n* = 11) compared with the normal group (9.44 ± 0.96 ng/mL; *n* = 11; *P* = 0.040; Fig. S4a), consistent with a compensatory, yet ultimately insufficient, anabolic signaling response in the cachectic state [27]. Serum IL‐6 was measured by ELISA in all 25 dogs; 24/25 samples yielded absorbance values below the lower detection limit of the assay (OD < 0.133), precluding quantitative analysis. A sensitivity analysis excluding C11 (IGF‐1 = 60.27 ng/mL, spleen tumor with reactive IGF‐1 elevation) further confirmed this finding (cachexia group excluding C11: 15.22 ± 1.74 ng/mL, *n* = 10 vs. normal: 9.44 ± 0.96 ng/mL, *n* = 11; *P* = 0.008), demonstrating the result is not driven by this single outlier case. In contrast, IGF‐1 did not differ significantly by cancer status alone (noncancer: 12.84 ± 2.07 ng/mL, *n* = 10 vs cancer: 16.13 ± 3.92 ng/mL, *n* = 12; *P* = 0.499; Fig. S4b).

**Table 1:** Demographic characteristics of patient dogs enrolled for this study.

Characteristics		Normal group (*n* = 13)	Cachexia group (*n* = 12)
Age (years, mean ± SD)		11.8 ± 2.8	13.3 ± 3.1
Sex	Male (Intact/Neutered)	6 (2/4)	5 (2/3)
	Female (Intact/Spayed)	7 (3/4)	7 (1/6)
BW (kg, mean ± SD)		6.2 ± 2.4	5.7 ± 2.3
Underlying Condition	Cancerous Disease	3	8
	Non‐Cancerous Disease	10	4

### 3.2 Clinical and radiographic assessment supports cachexia classification

The clinical classification of dogs into normal and cachexia groups was supported by radiographic evaluation of epaxial muscle mass. As shown in the representative images in Fig. 1B, dogs in the normal group exhibited considerable muscle volume dorsal to the thoracic spine (Fig. 1B). In contrast, dogs classified with cachexia displayed a marked reduction in this muscle mass, evidenced by a significantly narrowed space between the dorsal spinous processes and the skin, consistent with severe muscle atrophy (Fig. 1C).

### 3.3 A four‐miRNA signature demonstrates strong diagnostic performance for cachexia

**Fig. 2:**
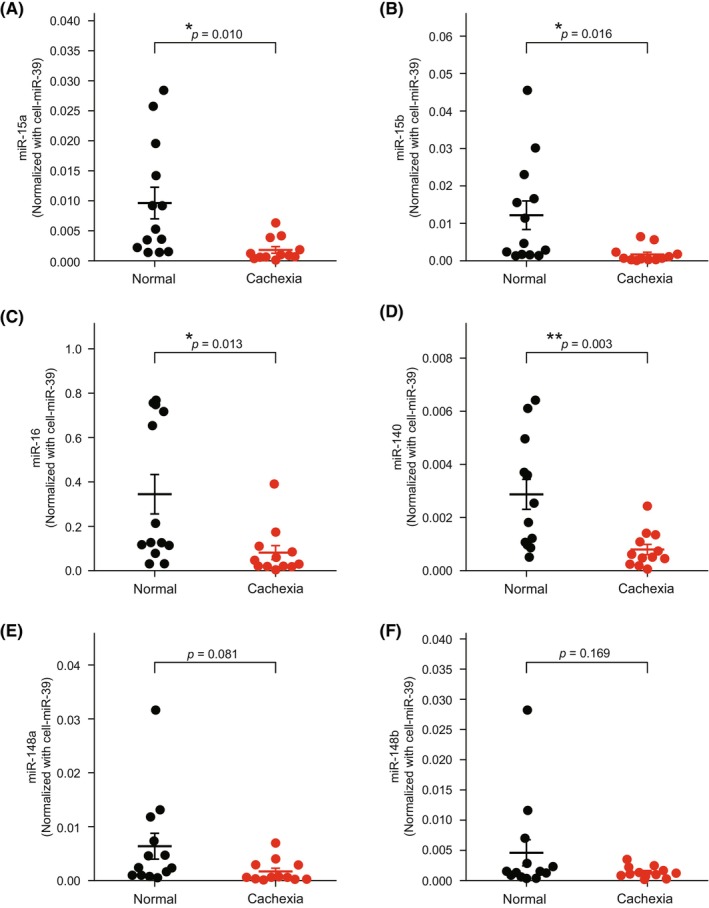
Circulating miRNA signatures are downregulated in dogs with cachexia. Relative expression levels of serum miR‐15a (A), miR‐15b (B), miR‐16 (C), miR‐140 (D), miR‐148a (E), and miR‐148b (F) in normal (*n* = 13) and cachexia (*n* = 12) groups, quantified by qRT‐PCR. Data are presented as box‐and‐whisker plots showing median, interquartile range, and min/max values. Expression was normalized to an exogenous spike‐in control (cel‐miR‐39). Statistical significance was determined by unpaired Student's *t*‐test. *P < 0.05, **P < 0.01. Each sample was assayed in technical triplicate; data points represent the mean of triplicate measurements per individual dog; group statistics are shown as mean ± SEM.

To identify potential biomarkers for muscle wasting, the serum levels of nine candidate miRNAs were quantified. This panel was selected based on a hypothesis‐driven approach targeting miRNAs with known roles in tumor suppression and muscle biology, making them relevant to the pathophysiology of cancer cachexia [21, 23, 28, 29, 30, 31]. This analysis revealed that a specific subset of these miRNAs was significantly altered in dogs with cachexia. Compared to the normal group, the cachexia group exhibited significantly lower circulating levels of miR‐15a (*P* < 0.05), miR‐15b (*P* < 0.01), miR‐16 (*P* < 0.05), and miR‐140 (*P* < 0.05) (Fig. 2A–D). The expression levels of the other five miRNAs investigated (miR‐148a, miR‐148b, miR‐152, miR‐195, and miR‐497) (Figs 2E,F and S1a–c, respectively) did not differ significantly between the two groups.

To evaluate the clinical utility of this downregulated miRNA signature, we performed ROC curve analysis. We first assessed the capacity of each of the four significantly altered miRNAs to function as an individual biomarker for cachexia. This analysis revealed a clear hierarchy of diagnostic performance (Table 2; Fig. S5a). Circulating miR‐16 was the most effective single biomarker for discriminating cachectic from normal dogs, achieving an excellent AUC of 0.899 (95% CI: 0.763–1.000; *P* < 0.001). MiR‐15b also demonstrated good performance (AUC = 0.815), while miR‐15a and miR‐140 showed fair discriminatory power (AUC = 0.720 and 0.714, respectively).

**Table 2:** Diagnostic performance of individual and panel biomarkers for cachexia.

miRNA	AUC	95% CI lower	95% CI upper	*P* (vs. AUC = 0.5)	Youden's J index	Optimal cutoff	Sensitivity	Specificity
miR‐15a	0.72	0.501	0.94	0.049	0.429	0.00184	0.75	0.679
miR‐15b	0.815	0.643	0.988	0.004	0.583	0.00231	0.833	0.75
miR‐16	0.899	0.763	1	< 0.001	0.75	0.07908	0.917	0.833
miR‐140	0.714	0.493	0.936	0.057	0.429	0.00108	0.667	0.762
4‐miRNA Panel^a^	0.923	0.806	1	< 0.001	0.762	0.46599	0.833	0.929

a The 4‐miRNA panel score was generated using a logistic regression model. While its AUC is numerically the highest, the 95% confidence interval overlaps substantially with that of miR‐16 alone, indicating that a statistically significant improvement in performance was not demonstrated in this cohort.

We next investigated whether combining these markers into a multi‐miRNA panel could further improve diagnostic accuracy. A composite biomarker score was generated for each subject using a logistic regression model with the expression levels of all four miRNAs as covariates. The resulting four‐miRNA panel yielded a numerically higher AUC of 0.923 (95% CI: 0.806–1.000) (Table 2). However, this result must be interpreted with significant caution. The logistic regression model was constructed using four predictor variables on a dataset with only 13 positive cases (cachexia), resulting in an events‐per‐variable (EPV) ratio of 3.25. This is below the recommended threshold to ensure model stability and prevent overfitting, which can lead to an artificially inflated performance estimate. The substantial overlap in the 95% confidence intervals between the panel (0.806–1.000) and miR‐16 alone (0.763–1.000) suggests that a statistically significant improvement in diagnostic performance was not demonstrated in this cohort. Therefore, while the panel concept is promising and warrants investigation in larger studies, miR‐16 stands out as the most robust single biomarker for cachexia identified in this study.

### 3.4 Circulating miR‐140 is a sex‐specific biomarker for cancer in female dogs

**Fig. 3:**
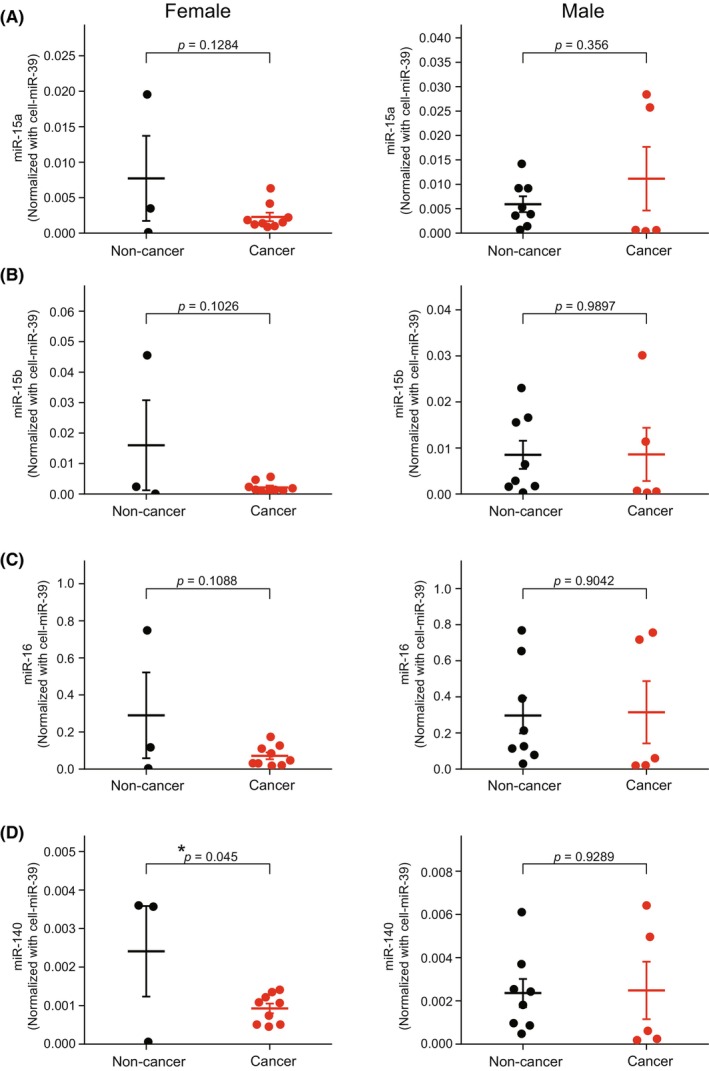
Circulating miR‐140 is a sex‐specific biomarker for cancer in female dogs. Relative expression of serum miR‐15a (A), miR‐15b (B), miR‐16 (C) and miR‐140 (D) in female dogs (Noncancer, *n* = 3; Cancer, *n* = 9; left panels) and male dogs (Noncancer, *n* = 8; Cancer, *n* = 5; right panels). Data are presented as box‐and‐whisker plots showing median, interquartile range, and min/max values. Expression was normalized to an exogenous spike‐in control (cel‐miR‐39). Statistical significance was determined by unpaired Student's *t*‐test. *P < 0.05. Each sample was assayed in technical triplicate; data points represent the mean of triplicate measurements per individual dog; group statistics are shown as mean ± SEM.

Given the strong association between cancer and cachexia in the cohort, we next investigated whether miRNA expression was altered in the context of cancer alone, independent of muscle wasting status. When the entire cohort was analyzed, no significant differences in the serum levels of any of the nine candidate miRNAs were observed between the cancer (*n* = 14) and noncancer (*n* = 11) groups (Fig. S2a–i, respectively). However, recognizing that sex can be a significant biological variable in cancer [32], a subsequent subgroup analysis stratified by sex was performed (Fig. 3A–D and Fig. S3a–e, respectively). This analysis revealed a striking, sex‐specific difference. Sex‐stratified expression of miR‐15a, miR‐15b, and miR‐16 in the female cohort showed a nonsignificant trend toward lower expression in the cancer subgroup (Fig. 3A–C, left panels, respectively), whereas the corresponding male comparisons showed no such trend (Fig. 3A–C, right panels). Notably, miR‐140 (Fig. 3D) revealed a striking, sex‐specific difference. Among female dogs, the serum level of miR‐140 was significantly downregulated in patients with cancer (*n* = 9) compared to those without cancer (*n* = 3) (*P* < 0.05) (Fig. 3D, left panel). In contrast, in male dogs there remained no significant difference in miR‐140 levels between the noncancer (*n* = 8) and cancer (*n* = 5) subjects (Fig. 3D, right panel).

The diagnostic potential of circulating miR‐140 as a sex‐specific cancer biomarker was assessed by ROC curve analysis in the female cohort (*n* = 12; cancer *n* = 9, noncancer *n* = 3). Upon verification of the ROC analysis against the raw data, the corrected AUC for miR‐140 in females is 0.667 (95% CI: 0.278–1.000; *P* = 0.045) (Table 3; Fig. S5b). At the optimal cutoff point determined by the Youden's J index (threshold ≤ 0.00141), miR‐140 achieved a sensitivity of 100% (9/9 cancer females detected) and a specificity of 66.7% (2/3 noncancer females correctly classified; J = 0.667). Importantly, the one noncancer female whose miR‐140 was misclassified below the threshold (C21, spayed female Schnauzer with severe cachexia from cardiac disease) had the lowest miR‐140 value in the entire cohort (0.000059). This finding, rather than weakening the analysis, provides direct *in vivo* evidence for the proposed dual‐hit suppression model: systemic inflammation from cachexia alone can suppress miR‐140 to near‐undetectable levels in the absence of cancer, while the combination of cachexia‐driven inflammation and tumor‐intrinsic ER*α* signaling in female cancer dogs produces a convergent, compounding suppression. These findings are preliminary, given the very small noncancer female subgroup (*n* = 3), and require validation in larger cohorts before miR‐140 can be recommended as a clinical cancer biomarker in female dogs.

**Table 3:** Diagnostic performance of individual and panel biomarkers for cancer in females.

miRNA	AUC	95% CI lower	95% CI upper	*P* (vs. AUC = 0.5)	Youden's J index	Optimal cutoff	Sensitivity	Specificity
miR‐15a	0.778	0.455	1	0.093	0.556	0.00139	1	0.556
miR‐15b	0.741	0.384	1	0.141	0.444	0.00134	1	0.444
miR‐16	0.704	0.33	1	0.197	0.444	0.019	1	0.444
miR‐140	0.667	0.278	1.000	0.045	0.667	0.00141	1.000	0.667
4‐miRNA Panel^a^	1	1	1	0.005	1	0.87195	1	1

a The AUC of 1.000 for the 4‐miRNA panel is a statistical artifact resulting from model overfitting (‘complete separation’) in a very small sample size (*n* = 12) with a high ratio of predictor variables to cases. This value is not indicative of true diagnostic performance and should be interpreted with extreme caution.

**Fig. 4:**
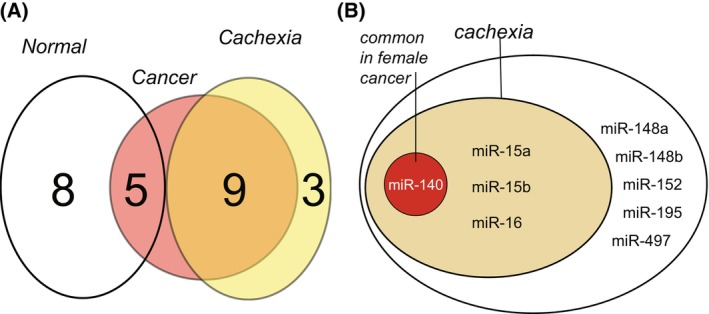
miRNA in pathological condition of sarcopenia and cancer. (A) Twenty‐five dogs are classified with two independent pathological conditions, cachexia or cancer. (B) Among nine candidate miRNAs, a four‐miRNA signature (miR‐15a, miR‐15b, miR‐16, and miR‐140) was identified for cachexia, reflecting systemic inflammation and catabolism. In parallel, miR‐140 was identified as a potent, female‐specific cancer biomarker, likely driven by tumor‐intrinsic hormone receptor signaling.

In summary, this study identified two distinct and clinically relevant circulating miRNA signatures from a single cohort of 25 senior dogs, whose distribution across the four clinical strata (normal/cachexia × noncancer/cancer) is shown in Fig. 4A. As illustrated schematically in Fig. 4B, the first signature was characterized by the downregulation of four miRNAs (miR‐15a, miR‐15b, miR‐16, and miR‐140) and was associated with cachexia, likely reflecting systemic inflammation and a failure of host anabolic signaling. Within this signature, miR‐16 emerged as the most robust individual biomarker. Second, in a sex‐specific context, miR‐140, as highlighted as the central node in Fig. 4B, was significantly lower in female dogs with cancer than in noncancer controls (*P* = 0.045). This sex‐specific downregulation was identified as a preliminary signal, grounded in the proposed dual‐hit suppression model, though discriminatory accuracy was modest in this pilot cohort (AUC = 0.667; noncancer females *n* = 3). The finding is potentially linked to tumor‐intrinsic hormone receptor signaling pathways and warrants prospective validation.

### 3.5 Post hoc analyses: Age confound and hormonal status

#### 3.5.1 Age‐miRNA correlations

Spearman correlation analyses across the full cohort (*n* = 25) identified significant negative associations between age and four miRNAs: miR‐15a (ρ = −0.520, *P* = 0.008), miR‐15b (ρ = −0.514, *P* = 0.009), miR‐16 (ρ = −0.475, *P* = 0.016), and miR‐497 (ρ = −0.636, *P* < 0.001) (Fig. S6a–d, respectively; Table S4). The remaining five miRNAs showed no significant age‐dependent variation.

#### 3.5.2 Deconvolution of age and cachexia effects on miR‐16

The mean age difference between the normal (11.77 ± 2.74 yr) and cachexia (13.92 ± 3.18 yr) groups was 2.15 years and was not statistically significant (*t* = −1.81, *P* = 0.083; Fig. S7a). In a multiple linear regression model including both age and cachexia status as simultaneous predictors (*n* = 25; Table 4), cachexia status remained a significant independent predictor of miR‐16 suppression (*β* = −0.228, 95% CI: −0.445 to −0.012, *P* = 0.040), while age was not (*β* = −0.016, *P* = 0.355; Fig. S7b). These results indicate that cachexia status, rather than chronological age, is the primary driver of miR‐16 suppression in this cohort.

**Table 4:** Multiple linear regression: Predictors of circulating miR‐16 expression (*n* = 25). Model: R^2^ = 0.271, Adjusted R^2^ = 0.204, df_residual = 22.

Variable	*β*	SE	*t*	*P*	95% CI
Intercept	0.536	0.213	2.511	0.020	[0.093, 0.979]
Age (years)	−0.016	0.017	−0.945	0.355	[−0.052, 0.019]
Cachexia status^a^	−0.228	0.104	−2.187	0.040*	[−0.445, −0.012]

a Cachexia status coded as binary variable: 0 = normal, 1 = cachexia.

* *P* < 0.05.

#### 3.5.3 Hormonal status sub‐analysis

Of the 25 dogs, 6 were gonadally intact and 19 were gonadectomized. Among female dogs, both intact and spayed individuals with cancer showed suppressed miR‐140 regardless of gonadal status (Fig. S8a), arguing against a circulating gonadal hormone‐dependent mechanism and supporting tumor‐intrinsic ER*α* signaling as the primary driver. Among male dogs, the two individuals with notably elevated miRNA expression (C08: intact, prostate tumor; C16: castrated, lipoma) belonged to the normal group, with distinct gonadal statuses that preclude a unified hormonal explanation (Fig. S8b). Their preserved miRNA levels are consistent with the finding that cancer alone, without concurrent cachexia, does not significantly alter circulating miRNA levels.

## 4 Discussion

The progression from healthy aging to a state of cachexia is a complex continuum involving changes in body composition. As illustrated in the supplementary conceptual diagram (Fig. S9), aging in dogs, starting from around 7 years, is often accompanied by a natural, gradual decline in muscle mass, function, and strength—a process known as sarcopenia [15, 33]. This age‐related muscle loss can be concurrent with an increase or maintenance of fat mass. Cachexia represents a more severe, disease‐driven acceleration of muscle wasting that is metabolically distinct from sarcopenia [15, 33]. However, they can co‐exist and are often difficult to distinguish clinically in their early stages [15]. Our study's focus on identifying biomarkers for cachexia addresses the critical need for tools that can objectively identify this pathological state of muscle loss beyond the expected effects of aging. This study provides compelling evidence for the utility of circulating miRNAs as noninvasive biomarkers for cachexia and cancer in a clinically relevant, spontaneous animal model. The principal findings are twofold: first, a distinct signature of four downregulated miRNAs (miR‐15a, miR‐15b, miR‐16, and miR‐140) is robustly associated with the clinical syndrome of cachexia in senior dogs. Second, circulating miR‐140 was identified as a novel, sex‐specific biomarker, with significantly lower levels observed in female dogs with cancer. These discoveries not only offer promising new avenues for veterinary diagnostics but also reinforce the value of comparative oncology in identifying conserved molecular pathways relevant to human disease.

A central finding is the significant systemic downregulation of miR‐15a, miR‐15b, and miR‐16 in the circulation of cachectic dogs, 75% of whom had an underlying malignancy. This observation is biologically profound, as the miR‐15/16 family members are canonical tumor suppressors whose loss is a hallmark of many human cancers [20, 21]. They function as a crucial homeostatic brake on cell proliferation by targeting anti‐apoptotic genes such as BCL2 [34]. Their systemic reduction in a cohort where cancer is highly prevalent is therefore consistent with a systemic failure to restrain tumorigenesis. Beyond cancer, these miRNAs are potent regulators of immune homeostasis; their loss can precipitate multi‐organ inflammation [22], a core driver of the hyper‐catabolic state in cachexia [5, 21, 35]. Thus, the observed decrease likely reflects a systemic failure of host control mechanisms, creating a permissive environment for both tumor progression and catabolism. This signature's relevance extends beyond inflammation, connecting directly to the molecular machinery of muscle wasting. The primary anabolic pathway in skeletal muscle is mediated by insulin‐like growth factor 1 (IGF‐1) and its downstream effectors, PI3K and AKT [36, 37]. Recent evidence has established a direct regulatory link between the miR‐15 family and this pathway. A long noncoding RNA, LncIRS1, acts as a ‘sponge’ for the miR‐15 family [23]. By sequestering miR‐15 members, LncIRS1 prevents them from suppressing their target, Insulin Receptor Substrate 1 (IRS1), a critical protein in the IGF‐1/PI3K/AKT signaling cascade [23]. This LncIRS1/miR‐15/IRS1 axis is a key regulator of myogenesis and a defense against muscle atrophy [23]. Therefore, the systemic reduction of the miR‐15/16 family in our cachectic cohort is mechanistically linked to the failure of the very anabolic pathways essential for maintaining muscle mass. Notably, our finding that circulating miR‐16 is downregulated in cancer‐associated cachexia contrasts with reports from mouse models of age‐related sarcopenia, which describe an upregulation of pathogenic, circulating exosomal miR‐16‐5p [38]. This apparent discrepancy reinforces the fundamental pathophysiological differences between these two muscle‐wasting syndromes [39]. Cachexia is a systemic, disease‐driven inflammatory condition, whereas sarcopenia is primarily an age‐related degenerative process [39]. The systemic downregulation of miR‐16 in our cachexia model likely reflects a global failure of host immune and tumor‐suppressive controls. In contrast, the upregulation of exosomal miR‐16 in sarcopenia models appears to function as a pro‐apoptotic signal broadcast from a site of local decay [38]. This suggests the direction of change of circulating miR‐16 is context‐dependent and may distinguish the systemic inflammatory nature of cachexia from the degenerative signaling of sarcopenia.

A notable finding of this study is the sex‐specific downregulation of circulating miR‐140 in female dogs with cancer (*P* = 0.045), though the corrected AUC (0.667) reflects the modest discriminatory ability achievable in this small pilot cohort (noncancer females *n* = 3). Critically, the mechanistic interpretation of this finding is strengthened, rather than weakened, by the data: the most severely cachectic noncancer female (C21) also exhibited profoundly suppressed miR‐140 (0.000059), providing direct *in vivo* evidence that systemic inflammatory signaling from cachexia alone can suppress miR‐140 independently of cancer. This supports the ‘dual‐hit’ suppression model: (1) a baseline suppressive pressure from cachexia‐driven inflammation (TNF‐*α*‐mediated miR‐140 repression) [29, 40], compounded by (2) tumor‐intrinsic ER*α*‐mediated transcriptional repression in hormone‐sensitive female cancers [24, 41]. The sex‐specificity of the overall pattern is biologically coherent: the female cancer cohort was predominantly composed of mammary gland and reproductive tract tumors (9/9 female cancer dogs), which are characteristically hormone receptor positive, providing the substrate for ER*α*‐mediated miR‐140 suppression. The finding remains preliminary and requires prospective validation in a larger, appropriately powered cohort with formal hormone receptor status profiling.

The female dogs in our cancer cohort predominantly presented with malignancies of the mammary gland and reproductive tract (Table S2), tumors that are frequently hormone receptor positive. This observation has a strong biological basis. In human breast cancer, miR‐140 is a well‐documented tumor suppressor [42, 43]. Mechanistic studies have demonstrated that ligand‐activated estrogen receptor alpha (ER*α*) binds directly to a specific estrogen response element (ERE) in the miR‐140 promoter, leading to potent transcriptional repression of this miRNA [24]. Therefore, the dramatic reduction of circulating miR‐140 observed exclusively in our female cancer cohort is likely a direct systemic readout of active, tumor‐intrinsic ER*α* signaling. This finding provides a powerful biological explanation for our observation and validates the domestic dog as a high‐fidelity model for studying the interplay between hormone signaling, miRNA dysregulation, and the progression of human female‐specific cancers.

The identification of miR‐140 as a key biomarker in both the general cachexia signature and the female‐specific cancer signature highlights its dual functionality. This can be explained by a ‘compounding effect’ model, where two distinct pathological processes converge to suppress its expression. First, the systemic inflammation that defines cachexia provides a baseline suppressive pressure on miR‐140. Cachexia is driven by pro‐inflammatory cytokines, most notably tumor necrosis factor‐alpha (TNF‐*α*) [40]. Studies in other cellular contexts have shown that inflammatory stimuli, including TNF‐*α* and interleukin‐1*β* (IL‐1*β*), actively suppress miR‐140 expression [41]. Furthermore, in a mouse model of endotoxin‐induced sepsis, upregulation of miR‐140 was shown to reduce serum levels of TNF‐*α* and protect against muscle atrophy [29]. This establishes a mechanistic link between the inflammation of cachexia and the moderate downregulation of miR‐140 in our overall cachectic group. Second, in female dogs with hormone‐sensitive cancers, this inflammation‐driven suppression is likely compounded by the potent, tumor‐intrinsic ER*α*‐mediated repression [24]. This ‘dual‐hit’ provides a mechanistically coherent explanation for why miR‐140 was most profoundly suppressed in this subgroup, even within a pilot cohort too small to establish formal diagnostic accuracy. In addition to these systemic effects, miR‐140 downregulation has direct relevance to muscle biology. Recent work has shown that inhibiting miR‐140‐5p can promote muscle satellite cell proliferation by de‐repressing its target, Pax7, thereby enhancing muscle regeneration [25]. The reduction of miR‐140 in cachexia could therefore represent a failed compensatory attempt by the host to stimulate myogenesis—an attempt overwhelmed by the powerful catabolic drivers of the syndrome.

The IGF‐1 data provide additional mechanistic context. Serum IGF‐1 was significantly elevated in the cachexia group (*P* = 0.040), a seemingly paradoxical finding that is increasingly recognized in cancer cachexia: advanced cancer can stimulate GH‐independent IGF‐1 production as a compensatory attempt to maintain anabolism that ultimately fails to prevent muscle wasting [27, 44]. The concurrent downregulation of the miR‐15 family, which directly targets IRS1, the primary downstream signal transducer of the IGF‐1 receptor [23], suggests that while the IGF‐1 ligand is present, the anabolic cascade is simultaneously disrupted at the postreceptor level by miR‐15‐mediated IRS1 suppression. This two‐pronged failure (elevated ligand, impaired signaling) provides a more complete picture of the anabolic resistance that characterizes cancer cachexia, and positions the circulating miR‐15 family as biomarkers of this signaling failure rather than simply of IGF‐1 deficiency.

A major strength of this study is its use of a comparative oncology approach, which leverages the spontaneously occurring diseases of companion animals to inform human health. Preclinical cachexia research has historically relied on rodent models with artificially induced tumors such as the C26 colon adenocarcinoma or Lewis lung carcinoma models [45, 46]. While invaluable, these models may not fully recapitulate the chronic and heterogeneous nature of human cachexia [46]. In contrast, companion dogs offer a superior model. Dogs develop cancer spontaneously within an intact immune system, share the human environment, and possess a more comparable metabolism [33, 46]. As an outbred population, they also have the genetic heterogeneity like human patients [33, 46]. The dog is not just a model but a patient, developing a cachexia syndrome with clinical and metabolic alterations that closely mirror the human condition [15, 33]. Identifying a robust biomarker signature in this high‐fidelity population can provide a powerful rationale for investigating the same signature in human cohorts. Therefore, the dog serves as a critical, spontaneous, and outbred model that bridges the gap between contrived rodent experiments and complex human clinical trials, potentially accelerating the development of both diagnostic tools and novel therapeutic strategies.

Still, this study has several limitations. The most significant is the modest sample size (*n* = 25), which impacts the statistical power of our analyses and necessitates caution in interpreting the results of the multi‐miRNA panels. The logistic regression models used to generate the panel scores were developed with low events‐per‐variable (EPV) ratios, a condition known to increase the risk of model overfitting and yield performance estimates with wide confidence intervals that reflect high statistical uncertainty. This was particularly evident in the female cancer sub‐analysis, where the panel's perfect AUC was a clear statistical artifact of the small sample size and severe class imbalance (Table 3). Consequently, while the panels are conceptually promising, their clinical utility cannot be confirmed without validation in much larger, independent, and prospectively collected cohorts. A further limitation is the substantial overlap between the cachexia and cancer cohorts, with a 75% prevalence of cancer in the cachectic group (Table S2). This unequal distribution introduces a potential confounding variable. Therefore, the four‐miRNA signature should be interpreted with caution, as it may more accurately represent cancer‐associated cachexia. Future studies are needed to validate this signature in dogs with cachexia from non‐neoplastic causes. Additionally, the predominance of small breeds (primarily Shih Tzu and Maltese) in this cohort limits the generalizability of the findings; future studies should enroll a broader spectrum of breed sizes, as large‐breed dogs have distinct cancer epidemiology and metabolic profiles that may influence circulating miRNA signatures. Finally, the cross‐sectional design identifies strong associations but does not establish causality or the predictive value of these miRNAs for cachexia onset.

Although Spearman correlation analyses identified significant negative associations between age and four miRNAs in this geriatric cohort (Section 3.5.1; Fig. S6), multiple linear regression demonstrated that cachexia status, not age, is the independent predictor of miR‐16 suppression after simultaneous covariate control (Section 3.5.2; Table 4). The 2.15‐year mean age difference between groups, while not statistically significant (*P* = 0.083), was not randomized and reflects the retrospective cohort design; future prospective studies must enroll age‐matched controls or formally model age as a covariate to isolate the disease‐specific miRNA signal. This design requirement is identified as the highest‐priority methodological consideration for translational validation in human cachexia cohorts.

## 5 Conclusion

In conclusion, this study, conducted in a translationally relevant spontaneous canine model, successfully identifies a circulating four‐miRNA signature (miR‐15a, miR‐15b, miR‐16, and miR‐140) as a promising, noninvasive biomarker panel for cancer‐associated cachexia. It identifies circulating miR‐140 as a candidate sex‐specific biomarker in female dogs, with a statistically significant group difference (*P* = 0.045) that is mechanistically grounded in estrogen receptor signaling and warrants prospective validation in larger, appropriately powered cohorts. These discoveries reinforce the molecular parallels between canine and human oncology and highlight the potential of comparative research to accelerate the development of diagnostic tools for both species.

The findings presented here provide a strong foundation for future research. First, validation of these biomarker panels in larger, independent, and multicenter canine cohorts is essential. Longitudinal studies are required to determine if changes in these miRNAs can predict cachexia onset, monitor disease progression, or assess response to therapy. Second, future studies must be designed to deconvolve the confounding effects of cancer and cachexia. This can be achieved by validating the four‐miRNA panel in dogs with cachexia arising from noncancer etiologies, such as congestive heart failure or chronic kidney disease, to determine if the signature is specific to the cachectic phenotype or is predominantly a marker of cancer‐driven disease. Third, mechanistic studies are warranted to confirm the proposed biological pathways in a canine‐specific context. For example, using canine mammary tumor cell lines to validate that estrogen receptor signaling directly suppresses miR‐140 expression would solidify its role as a surrogate for hormone receptor status. Similarly, investigating the LncIRS1/miR‐15/IRS1 axis in canine myoblast cell lines would confirm the proposed link to anabolic resistance. Finally, these findings open a compelling avenue for therapeutic development. The systemic downregulation of these tumor‐suppressive and anti‐atrophic miRNAs suggests that replacement therapy using synthetic miRNA mimics could represent a novel strategy to counteract muscle wasting or inhibit tumor progression in dogs, a concept with direct parallels to strategies under investigation in human oncology.

## Conflict of interest

The authors declare no conflicts of interest.

## Author contributions

Conceptualization: S‐S.P., K.A., K.H., and K‐S.J.; performed the experiments: S‐S.P. and G.K., and K.H.; data analysis and interpretation: S‐S.P., K.A., and K‐S.J.; writing‐original draft preparation: S‐S.P. and K.A.; writing‐ reviewing and editing: K.A., S‐N.C., H‐K.L., and K‐S.J.; funding acquisition: H‐K.L. and K‐S.J.; materials and resources: K.H. and K‐S.J.

## Supporting information


**Table S1.** Nogo‐A (RTN4) targeting miRNA prediction.
**Table S2.** Criteria for clinical classification of normal and cachexia groups.
**Table S3.** Summary of signalment, clinical diagnosis, and group assignments for the 25 dogs enrolled in the study.
**Table S4.** Spearman rank‐order correlations between age and circulating miRNA expression.
**Fig. S1.** Circulating miRNA signatures that are not downregulated in dogs with cachexia.
**Fig. S2.** Analysis of miRNA in serum from non‐cancer and cancer dogs.
**Fig. S3.** Analysis of additional circulating miRNAs in a sex‐specific manner for cancer status.
**Fig. S4.** Serum IGF‐1 concentrations by clinical group.
**Fig. S5.** Receiver operating characteristic (ROC) curves.
**Fig. S6.** Spearman rank correlation between age and circulating miRNA expression.
**Fig. S7.** Multiple linear regression and age distribution analysis for miR‐16.
**Fig. S8.** miR‐140 expression stratified by gonadal status and cancer status.
**Fig. S9.** Conceptual diagram of the progression from sarcopenia to cachexia.

## Data Availability

The data that support the findings of this study are available in the supporting information of this article. Additional raw data that support the findings of this study are available from the corresponding author [jeongks@dhu.ac.kr] upon reasonable request.
