# Supplementary material for: Circulating microRNA signatures of cachexia and cancer in Canis familiaris as a comparative oncology model for human disease
Source: Mol Oncol. 2026 Jul 22:10.1002/1878-0261.70293. Online ahead of print. doi: 10.1002/1878-0261.70293 (PMC13399023; doi:10.1002/1878-0261.70293)
Supplement: Supplementary file 1 — Table S1. Nogo‐A (RTN4) targeting miRNA prediction. Table S2. Criteria for clinical classification of normal and cachexia groups. Table S3. Summary of signalment, clinical diagnosis, and group assignments for the 25 dogs enrolled in the study. Table S4. Spearman rank‐order correlations between age and circulating miRNA expression. Fig. S1. Circulating miRNA signatures that are not downregulated in dogs with cachexia. Fig. S2. Analysis of miRNA in serum from non‐cancer and cancer dogs. Fig. S3. Analysis of additional circulating miRNAs in a sex‐specific manner for cancer status. Fig. S4. Serum IGF‐1 concentrations by clinical group. Fig. S5. Receiver operating characteristic (ROC) curves. Fig. S6. Spearman rank correlation between age and circulating miRNA expression. Fig. S7. Multiple linear regression and age distribution analysis for miR‐16. Fig. S8. miR‐140 expression stratified by gonadal status and cancer status. Fig. S9. Conceptual diagram of the progression from sarcopenia to cachexia. [file MOL2-9999-0-s001.docx]

**Supplementary Materials**

**for**

**Circulating microRNA signatures of cachexia and cancer in Canis familiaris as a comparative oncology model for human disease.**

**[Running Title]** Circulating miRNA biomarkers in canine cachexia and cancer

Soon-Seok Park^1),*^, Kyongman An^2),3),*^, Gyeonghwa Kim^4),*^, Soo-Nyun Choi^1)^, Hong-Ki Lee^5)^, Keun Hur^4)^, Kyu-Shik Jeong^1),6),7),¶^

^*^ These authors contributed equally to this work.

**^¶^ Corresponding author**

Professor, Kyu-Shik Jeong, D.V.M., Ph.D.

- Address: Department of Pet Industry, Adventure College, Daegu Haany University,
  1 Haanydaero, Gyeongsan City 38610, Gyeongsangbuk-Do, Republic of Korea
- Phone: (O)+82+53+819+1738, (CP)+82+10+8812+1258
- E-mail: [jeongks@dhu.ac.kr](mailto:jeongks@dhu.ac.kr)

**Supplementary figures and tables**

Table S1. Nogo-A (RTN4) targeting miRNA prediction

Table S2. Criteria for clinical classification of normal and cachexia groups.

Table S3. Summary of signalment, clinical diagnosis, and group assignments for the 25 dogs enrolled in the study.

Table S4. Spearman rank-order correlations between age and circulating miRNA expression

Figure S1. Circulating miRNA signatures that are not downregulated in dogs with cachexia.

Figure S2. Analysis of miRNA in serum from non-cancer and cancer dogs.

Figure S3. Analysis of additional circulating miRNAs in a sex-specific manner for cancer status.

Figure S4. Serum IGF-1 concentrations by clinical group.

Figure S5. Receiver operating characteristic (ROC) curves

Figure S6. Spearman rank correlation between age and circulating miRNA expression.

Figure S7. Multiple linear regression and age distribution analysis for miR-16.

Figure S8. miR-140 expression stratified by gonadal status and cancer status.

Figure S9. Conceptual diagram of the progression from sarcopenia to cachexia.

1. **Supplementary tables and figures**

**Table S1. Nogo-A (RTN4) targeting miRNA prediction.** Top-ranked predicted microRNA regulators of RTN4 (Nogo-A) in human (Gene ID: 57142) identified by bioinformatic target prediction analysis. miRNAs included in the candidate panel tested in this study are highlighted.

| **Rank (Human)** | **Target Score** | **miRNA (Human)** | **Canine Homolog** | **Included in Panel** | **Known Biological Role** |
| --- | --- | --- | --- | --- | --- |
| 6 | 94 | hsa-miR-497-3p | cfa-miR-497 | Yes | Tumor suppressor; cell cycle S-phase arrest |
| 22 | 82 | hsa-miR-15b-5p | cfa-miR-15b | Yes | Canonical tumor suppressor; BCL2 regulation; IGF-1/IRS1 axis |
| 23 | 82 | hsa-miR-152-3p | cfa-miR-152 | Yes | DNA methylation regulation; cancer suppression |
| 24 | 82 | hsa-miR-16-5p | cfa-miR-16 | Yes | Canonical tumor suppressor; BCL2; inflammation; myogenesis |
| 27 | 82 | hsa-miR-195-5p | cfa-miR-195 | Yes | Tumor suppressor; cell cycle arrest; apoptosis |
| — | — | hsa-miR-15a-5p | cfa-miR-15a | Yes (related family) | miR-15a/16-1 cluster; BCL2; canonical tumor suppressor |
| — | — | hsa-miR-140-5p | cfa-miR-140 | Yes (added a priori) | Tumor suppressor in breast cancer; ERα regulation; muscle satellite cells |
| — | — | hsa-miR-148a-3p | cfa-miR-148a | Yes (added a priori) | Glioblastoma suppressor; methylation |
| — | — | hsa-miR-148b-3p | cfa-miR-148b | Yes (added a priori) | Breast cancer suppressor; migration inhibitor |

miRNAs added to the panel based on established roles in muscle biology and cancer biology (not in the top prediction list) are indicated with '—' in Rank/Score. Target prediction analysis was performed using miRNA target prediction software (miRSystem) against the human and mouse RTN4 gene. Canine homologs were identified based on >95% mature sequence identity to the human miRNA.

**Table S2. Criteria for clinical classification of normal and cachexia groups.**

| **Groups** | **Muscle wasting** | **Weakness** | **Age-appropriate activity level** | **Have diseases** |
| --- | --- | --- | --- | --- |
| **Normal** | No | No | Normal | Yes |
| **Cachexia** | Yes | Yes | Infirmity/Bad | Yes |

**Table S3. Summary of signalment, clinical diagnosis, and group assignments for the 25 dogs enrolled in the study.** (yr, years; F, female; M, male; CM, castrated male; SF, spayed female; BW, body weight)

| **Case**  **#** | **Age**  **(yr)** | **Sex** | **Breed** | **BW**  **(kg)** | **Symptom** | **Cachexia Group** | **Cancer** |
| --- | --- | --- | --- | --- | --- | --- | --- |
| **Normal Group (n=13)** | | | | | | | |
| C1 | 13 | F | shihtzu | 4.7 | Chronic dermatitis, Otitis externa, Cataract | No | No |
| C3 | 14 | M | shihtzu | 6 | Gastroenteritis (CRP 81) | No | No |
| C6 | 12 | CM | shihtzu | 6.1 | Perianal Adenoma | No | No |
| C7 | 12 | CM | schnauzer | 8.2 | Bladder Stones | No | No |
| C8 | 11 | M | dachshund | 11.7 | Prostate Tumor, Bladder Stones | No | Yes |
| C10 | 11 | SF | Y.terrier | 4 | Ovarian Cyst, Endometritis (Ovariohystorectomy), Mastitis | No | Yes |
| C12 | 11 | SF | mix | 5.8 | Tail Gland tumor, Mammary Gland Tumor | No | Yes |
| C13 | 7 | CM | shihtzu | 5.6 | Fracture in Mandible | No | No |
| C16 | 16 | CM | shihtzu | 7.5 | Tail Gland tumor. Multiple Giant Lipoma | No | Yes |
| C17 | 8 | CM | shihtzu | 6.4 | Left Hip Osteoarthritis | No | No |
| C18 | 16 | F | shihtzu | 2.8 | Megacolon, Ovarian Tumor (Ovariohystorectomy) | No | Yes |
| C19 | 13 | SF | mix | 5.4 | Cruciate Ligament Rupture | No | No |
| C28 | 9 | CM | pomeranian | 4.5 | Neuronergic Lameness | No | No |
| **Cachexia Group (12)** | | | | | | | |
| C2 | 8 | SF | maltese | 3.7 | Ovarian Cyst, Endometritis (Ovariohystorectomy) . Periodontitis | Yes | Yes |
| C11 | 11 | CM | maltese | 5.5 | Chronic Dermatitis, Severe Itching, Spleen Tumor | Yes | Yes |
| C21 | 15 | SF | schnauzer | 6.4 | Heart Disease. Cardiac Hypertrophy, Ascites | Yes | No |
| C22 | 16 | M | shihtzu | 5 | Cerebral Infarction.Degenerative Brain Atrophy. Otitis Media, Cognitive Dysfunction, Convulsion | Yes | No |
| C23 | 15 | SF | schnauzer | 6.7 | Melanoma in Mandible, Cachexia | Yes | Yes |
| C24 | 16 | SF | mix | 3.1 | Thoracic tumor, Intrahepatic Tumor, Heart Disease (SYNCOPE). Degenerative Arthritis.Gingivitis | Yes | Yes |
| C26 | 13 | CM | C.spaniel | 10.6 | Degenerative Arthritis, Mid-Renal Failure Anasplasmosis | Yes | No |
| C29 | 15 | SF | shihtzu | 5.5 | Mammary Gland Tumor, Neurogenicity Lameness | Yes | Yes |
| C31 | 10 | SF | schnauzer | 5.5 | Spleen Tumor Rupture, Severe Anemia., Hemangiomasarcoma | Yes | Yes |
| C32 | 19 | CM | c.spaniel | 8.8 | Degeneration Arthritis,C cognitive Dysfunction, Skin Tumor | Yes | Yes |
| C33 | 12 | CM | maltese | 3.5 | Lameness. Vomiting, Anorexia. Hypoadrenalism (Addison's disease) | Yes | No |
| C34 | 17 | F | shihtzu | 4.2 | Pyometra (Ovariohystorectomy) Mammary Gland Tumor, Adrenal Tumor | Yes | Yes |

**Table S4. Spearman rank-order correlations between bge and circulating miRNA expression (n=25).**

| **miRNA** | **Spearman ρ** | **P-value** | **Significance** |
| --- | --- | --- | --- |
| miR-15a | −0.520 | 0.008 | ** |
| miR-15b | −0.514 | 0.009 | ** |
| miR-16 | −0.475 | 0.016 | * |
| miR-497 | −0.636 | <0.001 | *** |

*P<0.05, **P<0.01, ***P<0.001.

1. **Supplementary figures and legends**

**
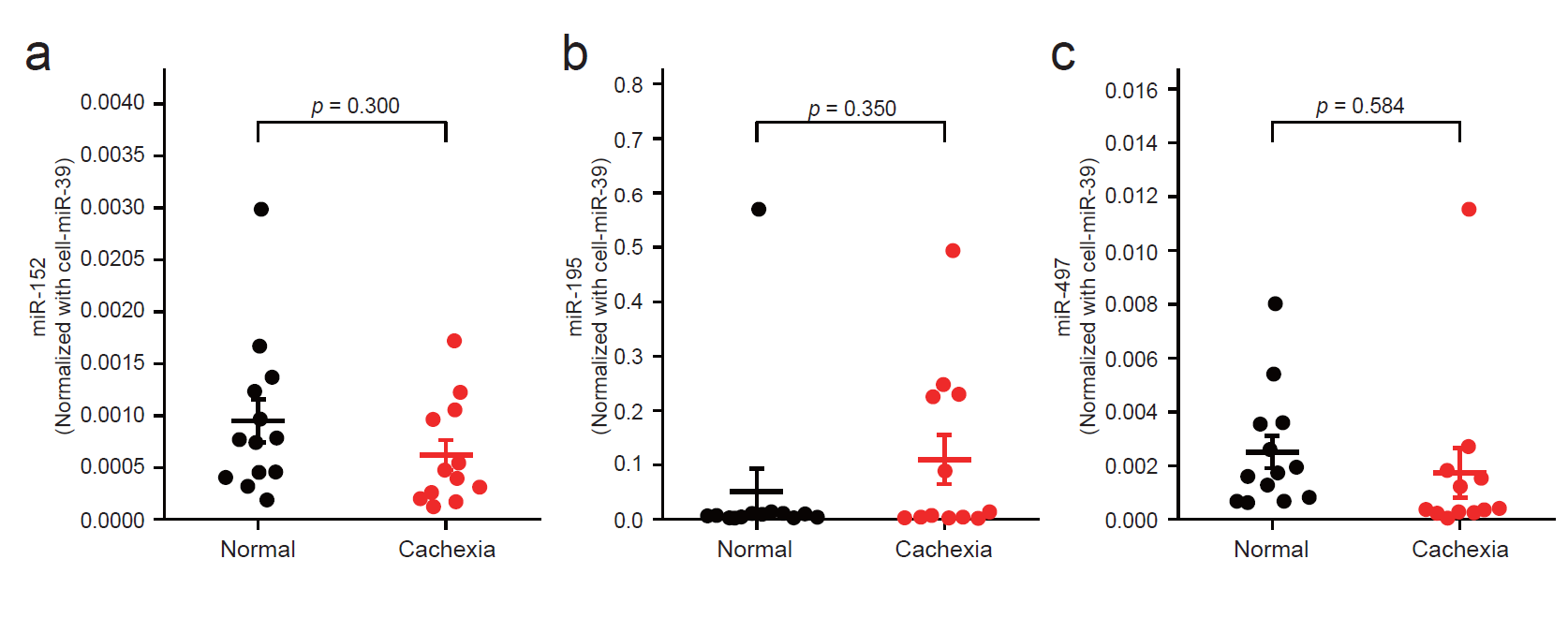
**

**FIGURE S1. Circulating miRNA signatures that are not downregulated in dogs with cachexia.**

Relative expression levels of serum miR-152 (**a**), miR-195 (**b**), and miR-497 (**c**) in normal (n=13) and cachexia (n=12) groups, quantified by qRT-PCR. Data are presented as box-and-whisker plots showing median, interquartile range, and min/max values. Expression was normalized to an exogenous spike-in control (cel-miR-39). Statistical significance was determined by unpaired Student’s t-test. **P*<0.05, ***P*<0.01. Each sample was assayed in technical triplicate; data points represent the mean of triplicate measurements per individual dog; group statistics are shown as mean ± SEM.

**
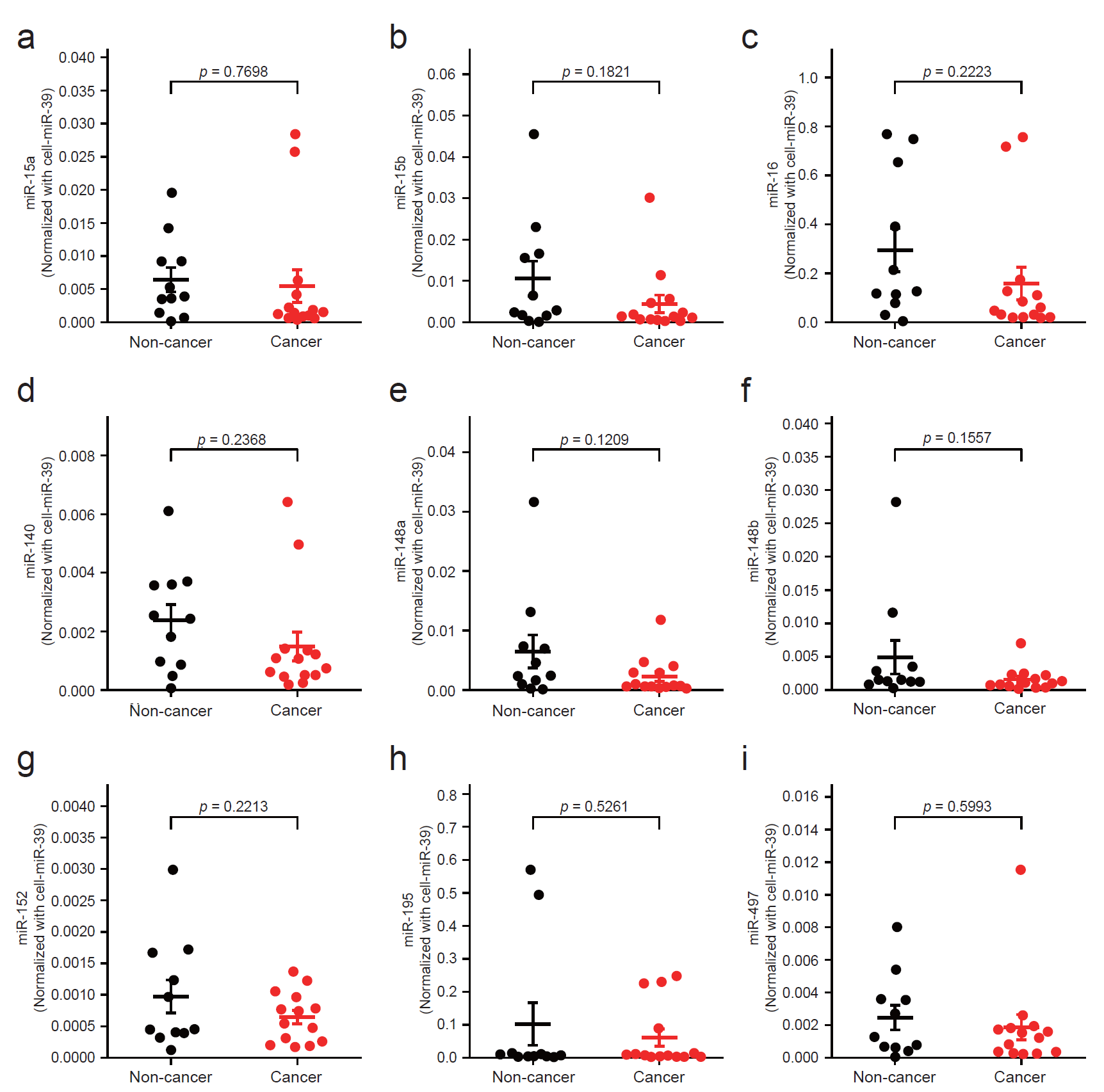
**

**Figure S2. Analysis of miRNA in serum from non-cancer and cancer dogs.**

Relative expression levels of serum miR-15a (**a**), miR-15b (**b**), miR-16 (**c**), miR-140 (**d**), miR-148a (**e**), miR-148b (**f**), miR-152 (**g**), miR-195 (**h**), and miR-497 (**i**) in non-cancer (n=11) and cancer (n=14) groups, quantified by qRT-PCR. Data are presented as box-and-whisker plots showing median, interquartile range, and min/max values. Expression was normalized to an exogenous spike-in control (cel-miR-39). Statistical significance was determined by unpaired Student’s t-test. Each sample was assayed in technical triplicate; data points represent the mean of triplicate measurements per individual dog; group statistics are shown as mean ± SEM.

**
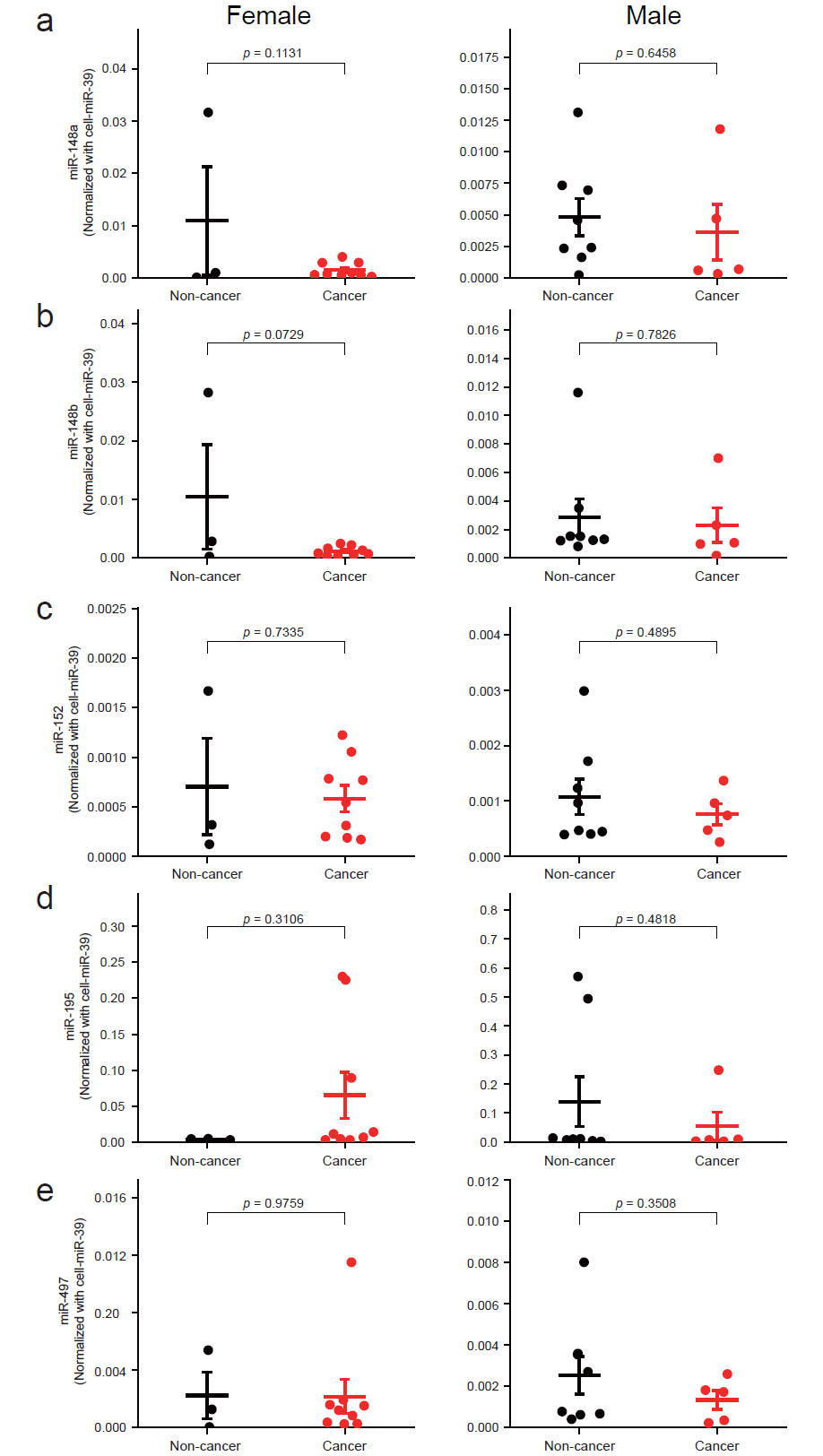
**

**Figure S3. Analysis of additional circulating miRNAs in a sex-specific manner for cancer status.**

Relative expression of serum miR-148a (**a**), miR-148b (**b**), miR-152 (**c**), miR-195 (**d**) and miR-497 (**e**) in female dogs (non-cancer, n=3; Cancer, n=9; left panels) and male dogs (non-cancer, n=8; cancer, n=5; right panels). Data are presented as box-and-whisker plots showing median, interquartile range, and min/max values. Expression was normalized to an exogenous spike-in control (cel-miR-39). No statistically significant differences were observed (unpaired Student’s t-test). Each sample was assayed in technical triplicate; data points represent the mean of triplicate measurements per individual dog; group statistics are shown as mean ± SEM.


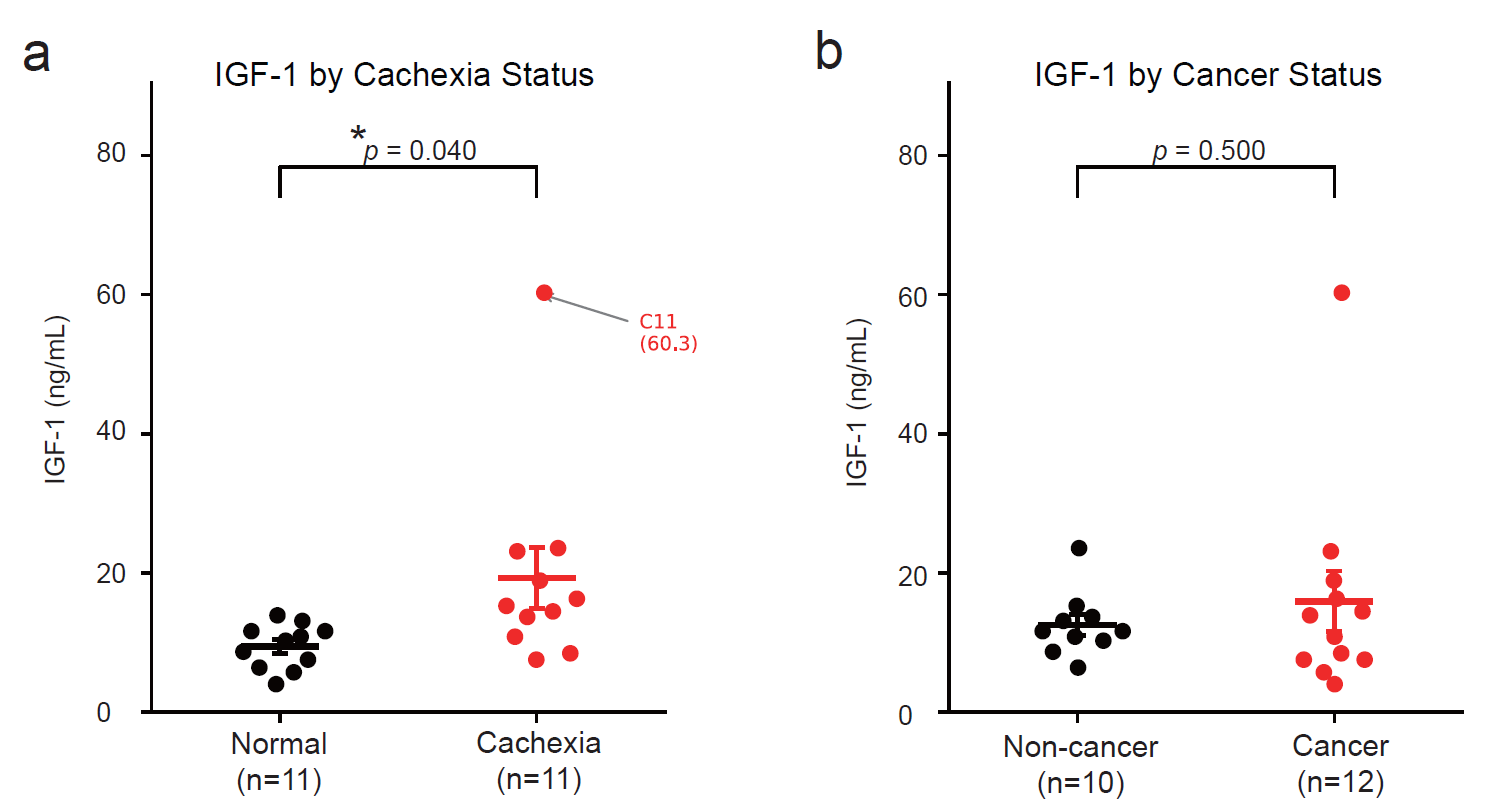


**Figure S4. Serum IGF-1 concentrations by clinical group.**

(a) IGF-1 levels in normal (n=11) and cachexia (n=11) groups. Data shown as individual values with mean ± SEM. C02's IGF-1 (256.5 ng/mL) was excluded as a hormonal artifact (active bilateral ovarian cysts); all other C02 data were retained. Significantly elevated IGF-1 in the cachexia group (*P*=0.040, Student's t-test) is consistent with compensatory anabolic signaling that is ultimately insufficient to prevent muscle loss. (b) IGF-1 levels in Non-cancer (n=10) and Cancer (n=12) groups. No significant difference was observed (*P*=0.499). *P*<0.05 (*). Each sample was assayed in technical triplicate; data points represent the mean of triplicate measurements per individual dog; group statistics are shown as mean ± SEM.


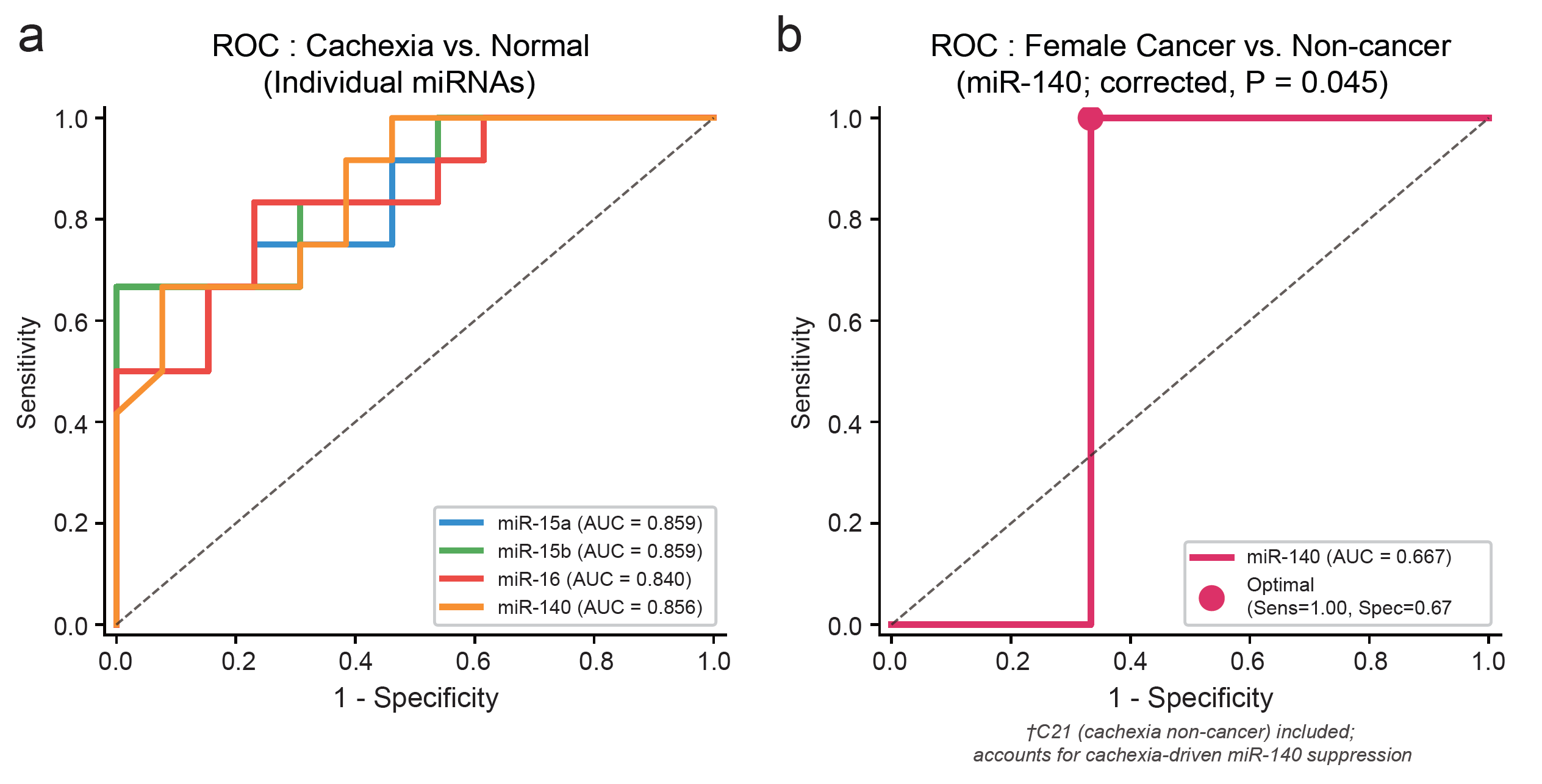


**Figure S5. Receiver operating characteristic (ROC) Curves**

(a) ROC curves for the four significantly altered miRNAs (miR-15a, -15b, -16, and -140) for discrimination between cachexia (n=12) and normal (n=13) groups. All four miRNAs demonstrate AUC values above the no-discrimination diagonal (AUC=0.5). (b) ROC curve for miR-140 in the female subgroup analysis (cancer n=9 vs. non-cancer n=3). The corrected AUC=0.667 (95% CI: 0.278–1.000; *P*=0.045) is displayed. The optimal Youden's J threshold (≤0.00141) is marked with a filled circle (Sensitivity=1.000, Specificity=0.667). A footnote indicates that the non-cancer group includes C21, a cachectic non-cancer female whose miR-140 suppression reflects cachexia-driven inflammation (see Discussion).


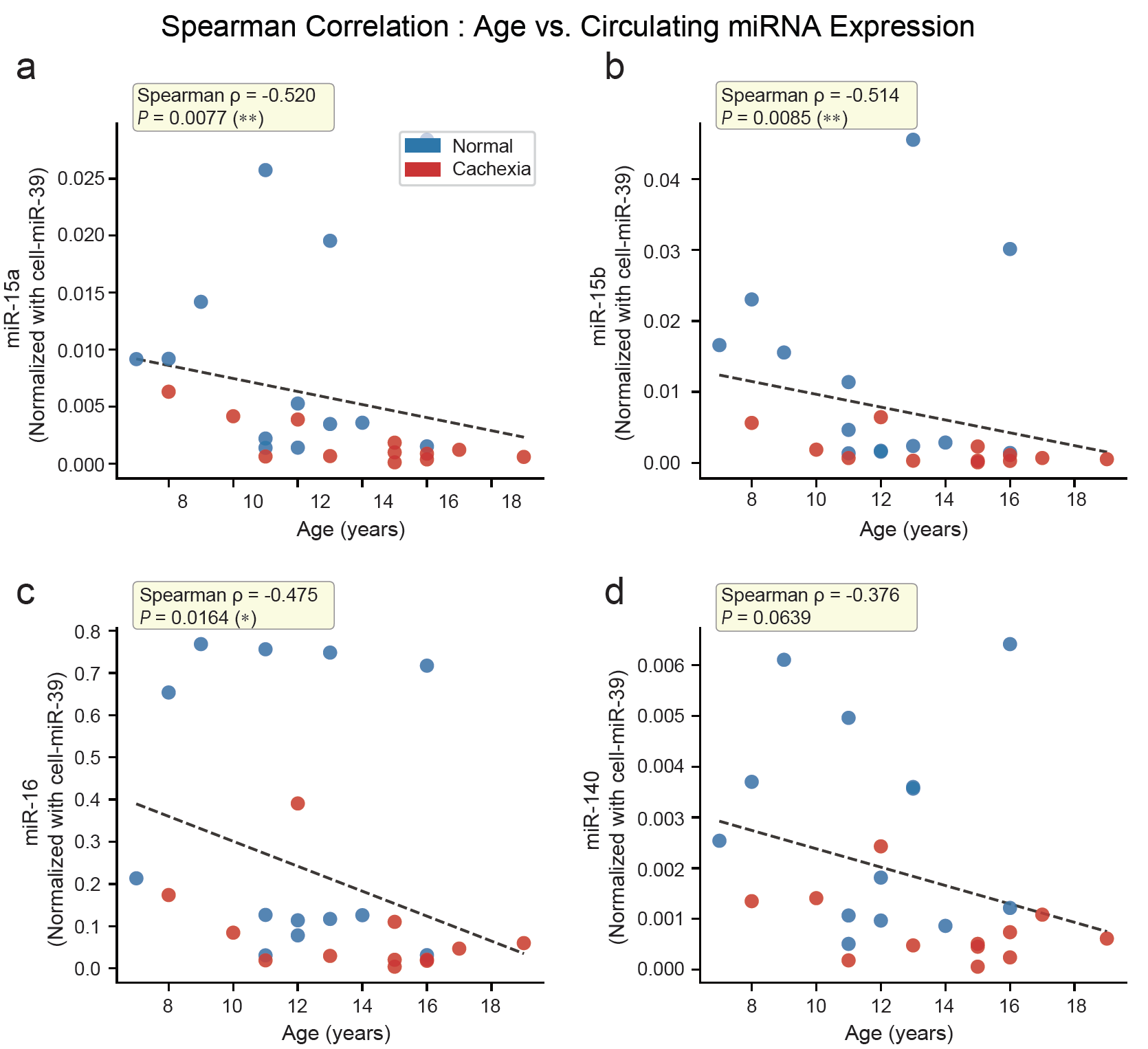


**Figure S6. Spearman rank correlation between age and circulating miRNA expression.**

Scatter plots showing the relationship between age (years) and relative expression of (a) miR-15a, (b) miR-15b, (c) miR-16, and (d) miR-140, in all 25 dogs. Points are colored by cachexia status (blue = normal; red = cachexia). Dashed lines represent linear regression fit. Spearman correlation coefficient (ρ) and P-value are shown for each panel. Significant negative correlations with age (*P*<0.05) were observed for miR-15a (ρ=−0.520, *P*=0.008), miR-15b (ρ=−0.514, *P*=0.009), and miR-16 (ρ=−0.475, *P*=0.016), indicating that age is a potential confounding variable that should be controlled in future studies.

**
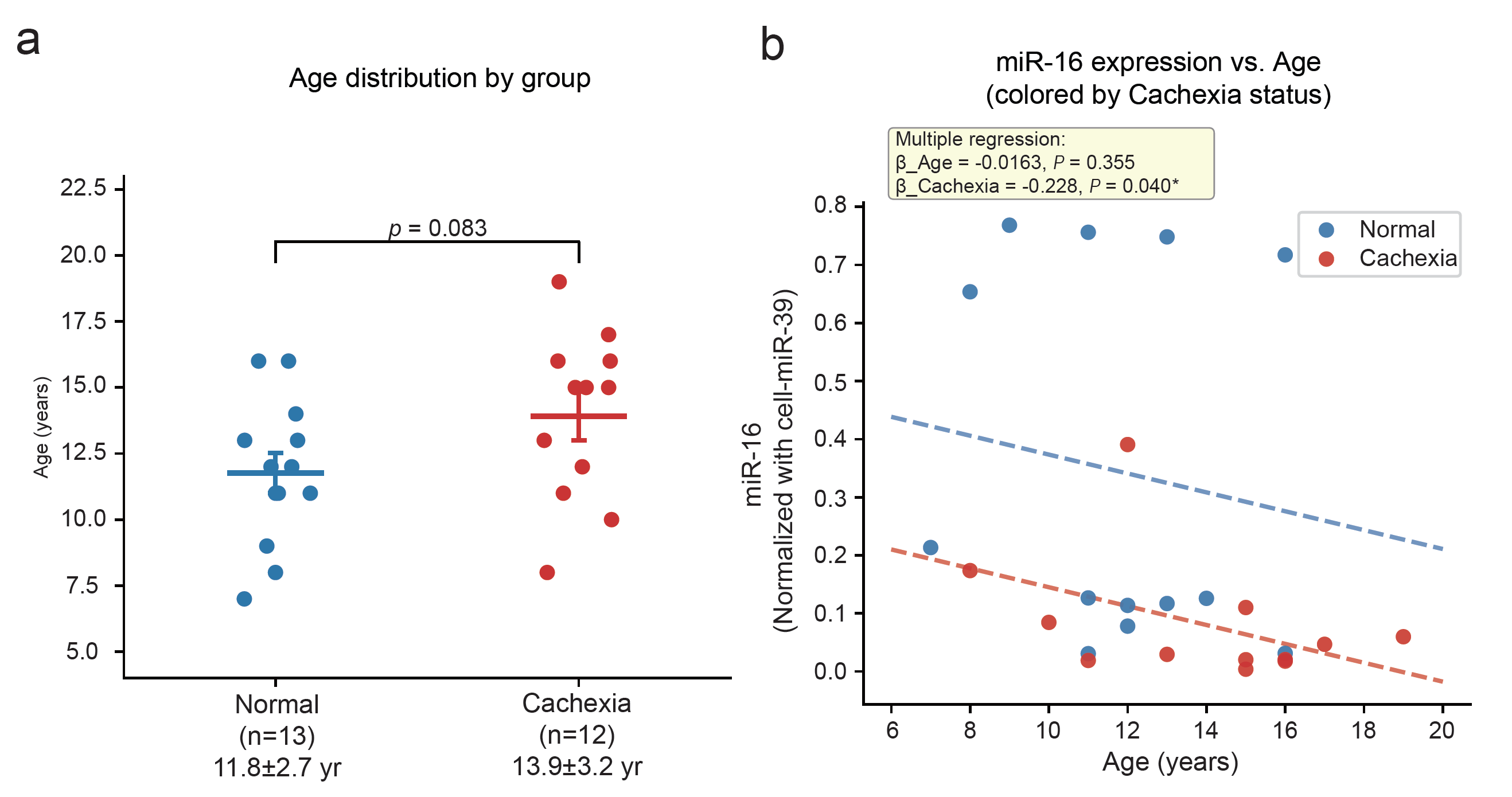
**

**Figure S7. Multiple linear regression and age distribution analysis for miR-16.** (a) Age distribution by group shown as dot plots with median and interquartile range. The 2.15-year mean age difference was not statistically significant (unpaired t-test: *P*=0.083). (b) Scatter plot of serum miR-16 expression (2^−ΔCt^, normalized to cel-miR-39) versus age (years) for normal (blue circles, n=13) and cachexia (red circles, n=12) groups. Partial regression lines from the multiple linear regression model (miR-16 ~ Age + cachexia_status) are shown separately for each group, illustrating that cachexia status, not age, drives the between-group difference in miR-16. Together, the two panels support the conclusion that the miR-16 suppression is a disease-driven, rather than age-driven, phenomenon.


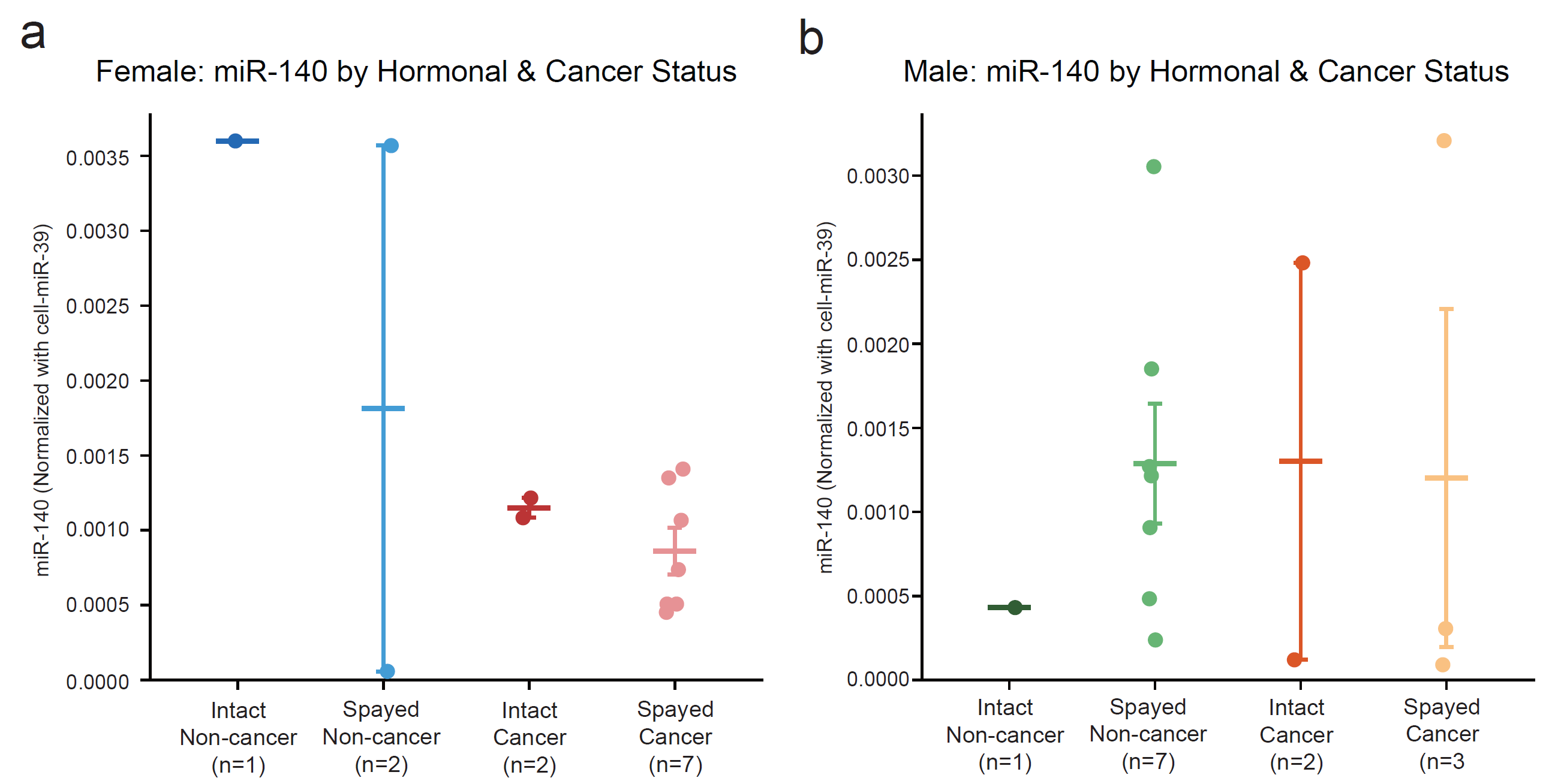


**Figure S8. miR-140 expression stratified by gonadal status and cancer status.**

(a) Female dogs: miR-140 expression in intact females (F) and spayed females (SF), stratified by cancer status. (b) Male dogs: miR-140 expression in intact males (M) and castrated males (CM), stratified by cancer status. Individual data points and mean ± SEM are shown. Subgroup sizes are small (n<5 per group), precluding formal statistical comparisons. No definitive pattern attributable to gonadal status alone was detected; the low miR-140 in female cancer dogs appears consistent across both intact and spayed animals, supporting a cancer-intrinsic (rather than gonad-dependent) suppression mechanism. A larger cohort with intact animals is required to definitively test the role of hormonal status.

**
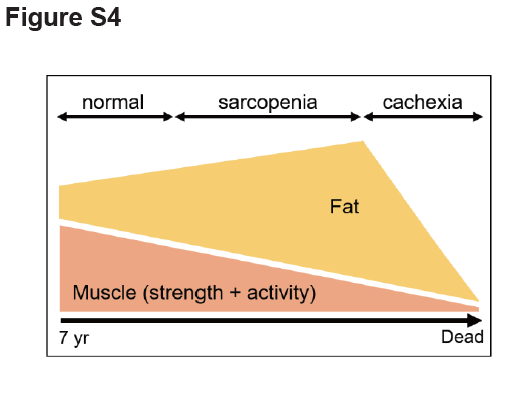
**

**Figure S9. Conceptual diagram of the progression from sarcopenia to cachexia.**

Conceptual illustration of the relationship between aging, sarcopenia, and cachexia in dogs. Sarcopenia is characterized by a gradual, age-related decline in muscle mass and function. Cachexia represents a more severe, disease-driven pathological state of muscle wasting, often superimposed on sarcopenia, that is driven by systemic inflammation and metabolic dysregulation.
